# Supplementary figures and images for: The Muscleblind-like protein MBL-1 regulates microRNA expression in Caenorhabditis elegans through an evolutionarily conserved autoregulatory mechanism
Source: PLoS Genet. 2023 Dec 22;19(12):e1011109. doi: 10.1371/journal.pgen.1011109 (PMC10773944; doi:10.1371/journal.pgen.1011109)

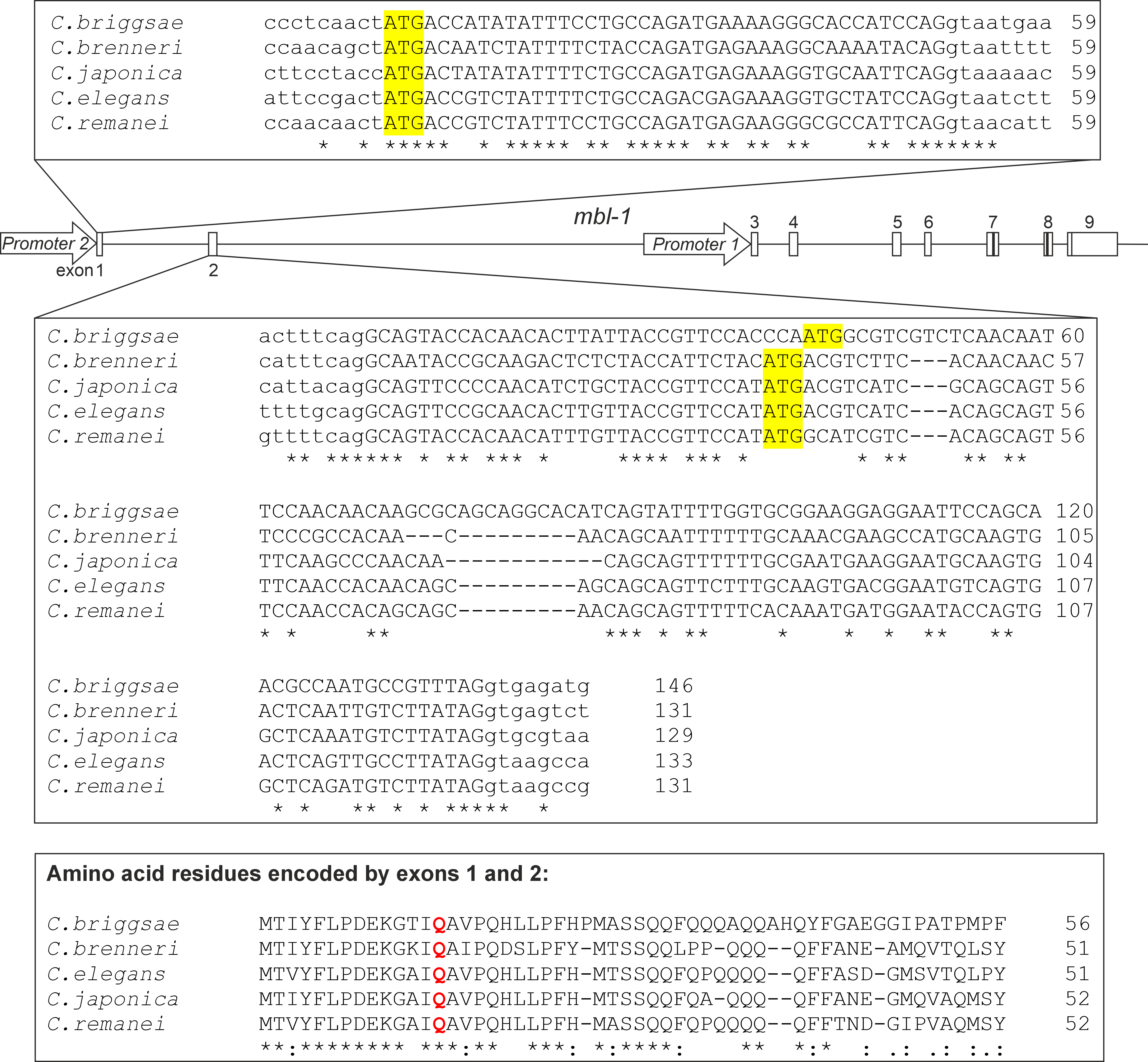

Supplement: S1 Fig — Depiction of the mbl-1 genomic region and DNA alignment of the genomic sequence surrounding exons 1 and 2 in the nematode lineage. Non-coding sequences are in lower case, coding sequences in upper case with potential start codons highlighted in yellow. Lower box depicts alignment of amino acid sequence encoded by exons 1 and 2, with the last amino acid encoded on exon 1 depicted in red. Asterisks indicate that the amino acid or nucleotide is the same for all sequences; a colon indicates that amino acids have similar chemical properties. (TIF) [file pgen.1011109.s001.tif]

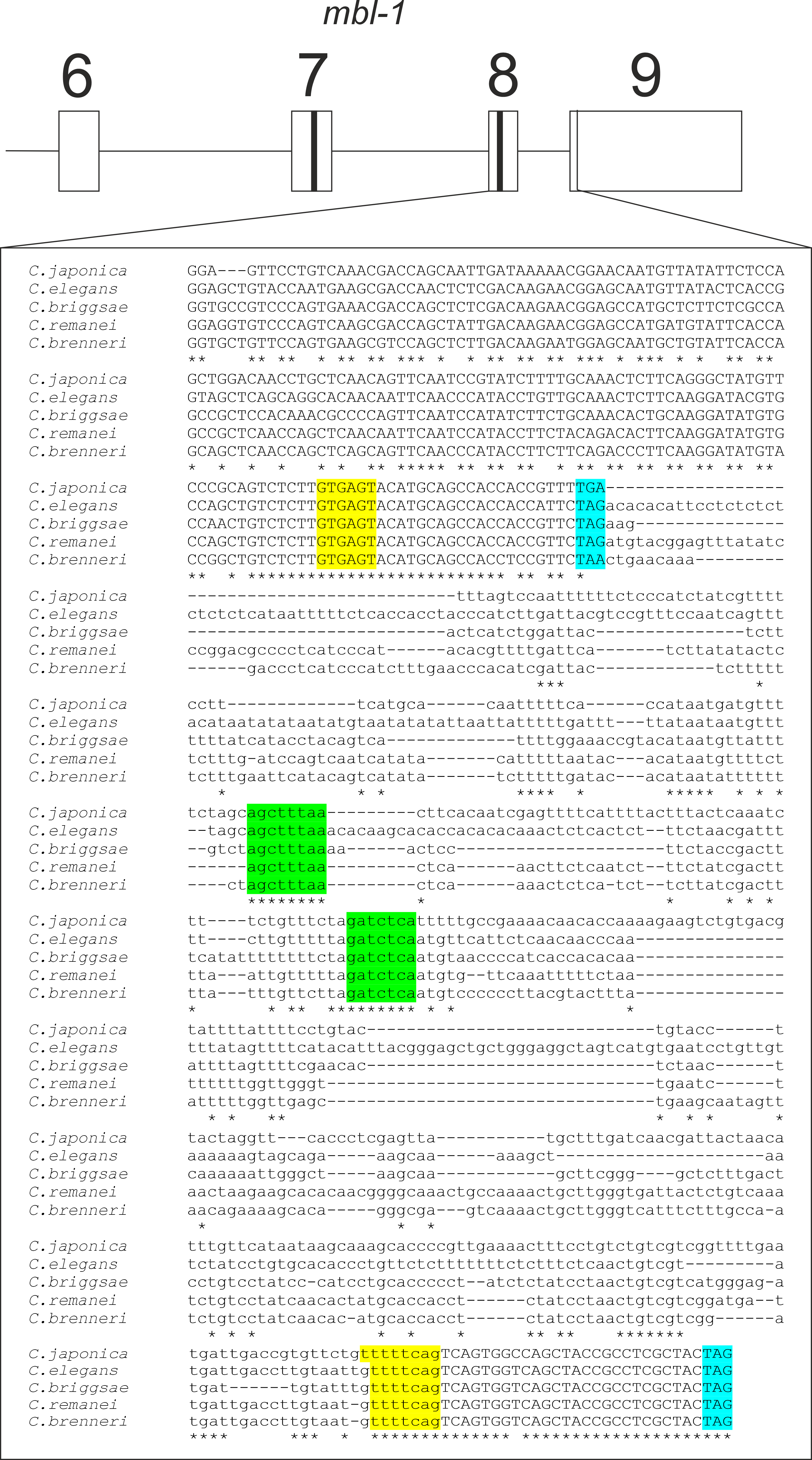

Supplement: S2 Fig — Depiction of the mbl-1 terminal exons and 3′-UTRs, and DNA alignment in the nematode lineage. Non-coding sequences are in lower case, and coding sequences in upper case. Highlighted in yellow are respectively the 5′ splice site and 3′ splice site of the alternative splicing event. Stop codons are highlighted in blue, conserved miRNA binding sites in green (miR-79 and miR-58/80/81/82, respectively). Asterisks indicate that the nucleotide is the same for all sequences. (TIF) [file pgen.1011109.s002.tif]

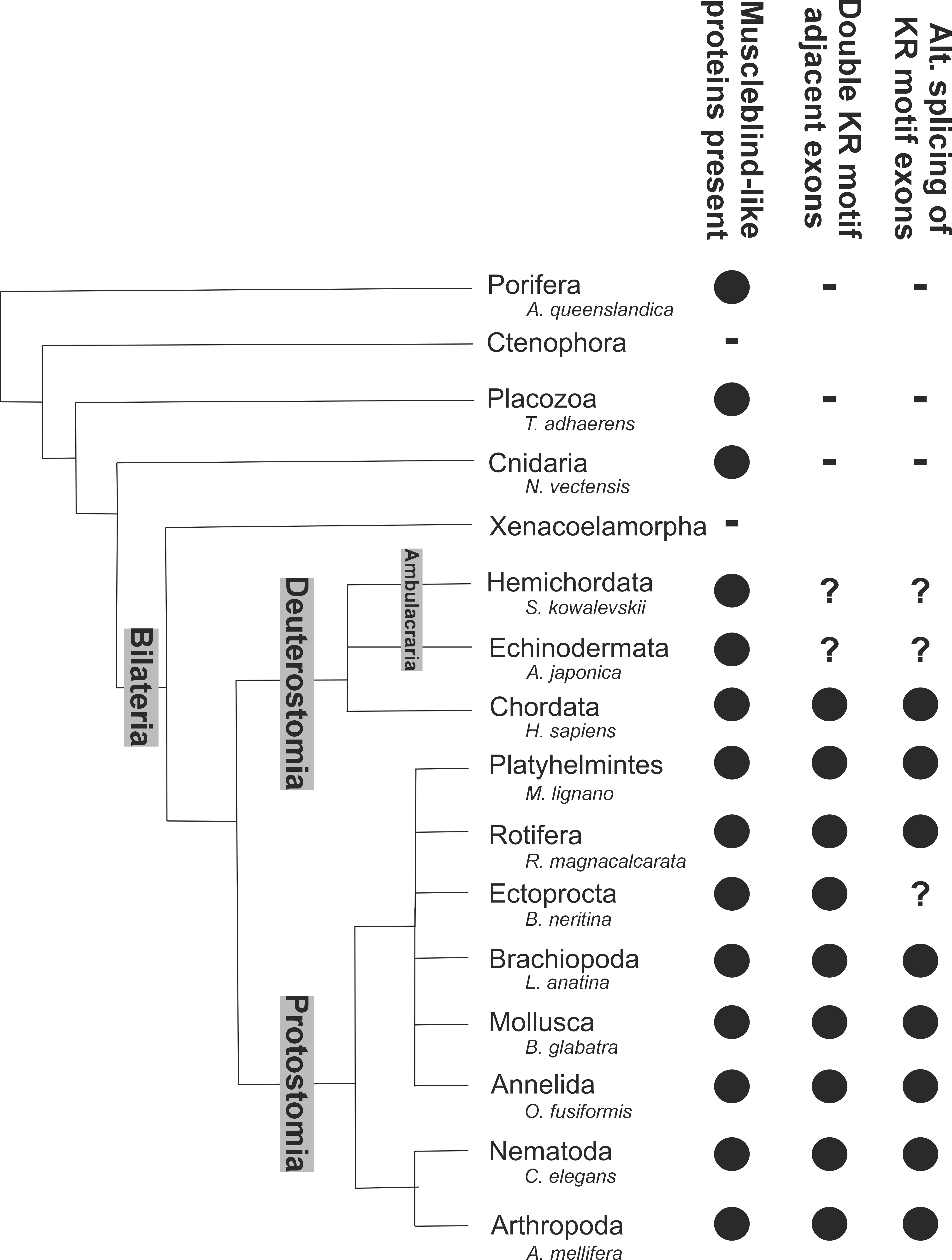

Supplement: S3 Fig — Phylogenetic representation of the presence of MBNL proteins within the animal kingdom, the double KR motif in two neighboring exons, and annotated alternative splicing associated with the KR motifs. Dots represent presence, hyphens absence, and question marks unidentified. (TIF) [file pgen.1011109.s003.tif]

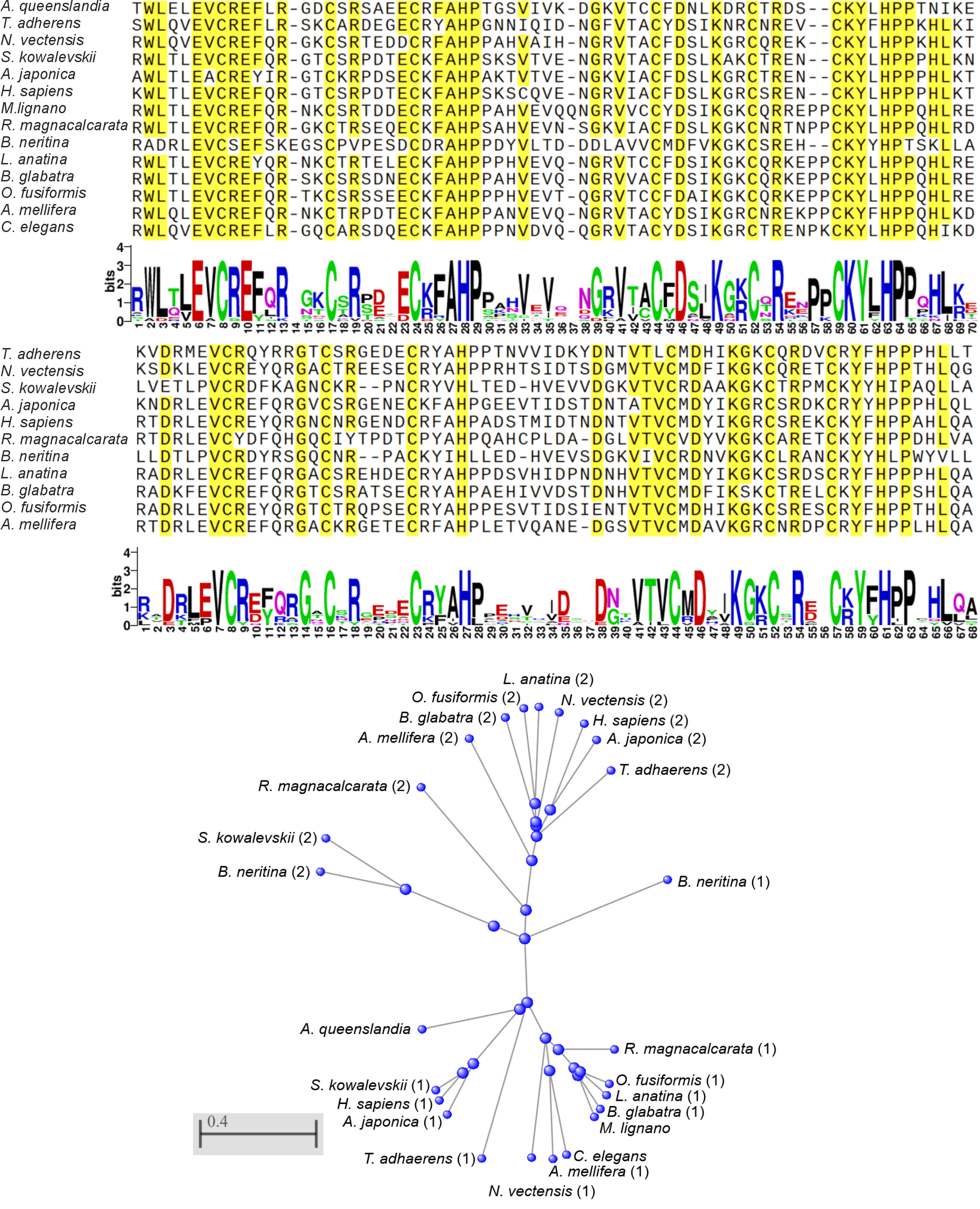

Supplement: S4 Fig — Protein alignment of TZF motifs 1 (top) and 2 (bottom). Amino acid sequence conservation > 85% is highlighted in yellow. Unrooted radiation tree depicts phylogenetic relationship between TZF motifs 1 (1) and 2 (2). The scale bar indicates 0.4 (40%) genetic variation for the length of the scale. (TIF) [file pgen.1011109.s004.tif]

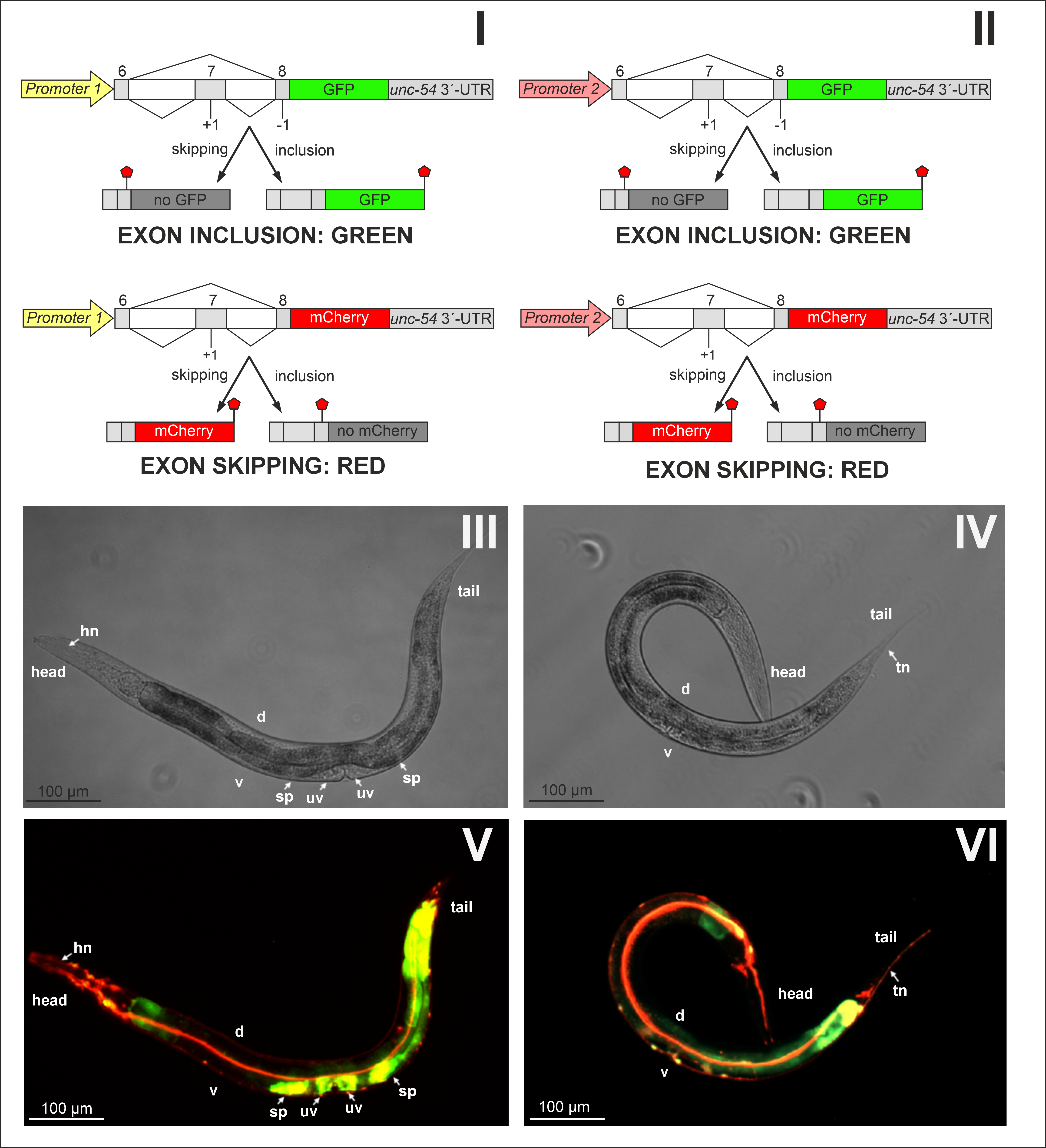

Supplement: S5 Fig — DIC and fluorescence images taken at the L4 stage of alternative splicing reporter animals for which GFP and mCherry genes were expressed under promoter 1 (panels I, III and V) or promoter 2 (II, IV and V), respectively. Arrows identify regions of differential expression; hn: unidentified sensory head neurons with dendrites on the dorsal site, sp: spermathecal, uv: unidentified cells in vulval region (putative uterine muscle cells), tn: unidentified tail neuron. (TIF) [file pgen.1011109.s005.tif]

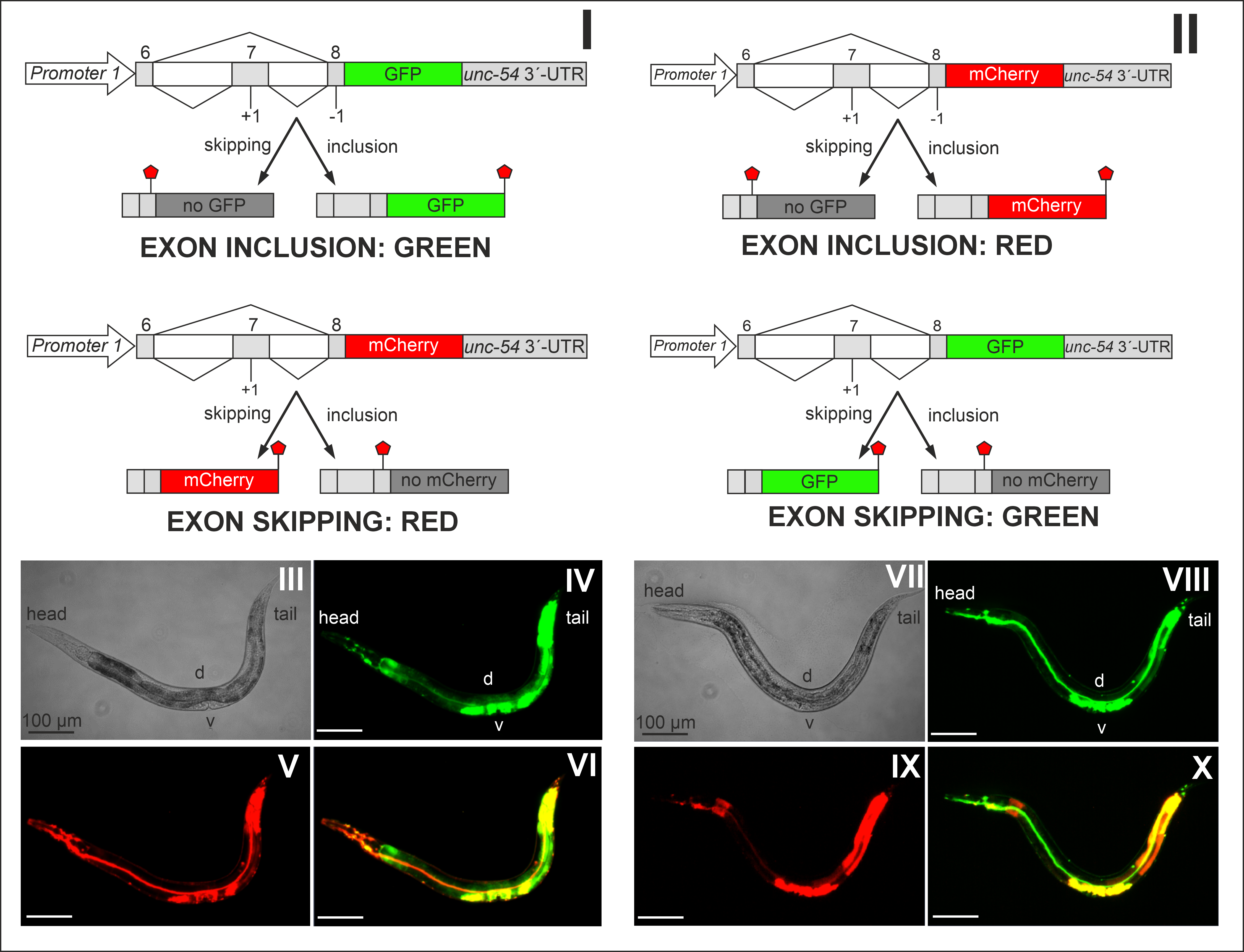

Supplement: S6 Fig — DIC and fluorescence images of alternative splicing reporter animals for which GFP and mCherry genes were switched (panels VII to X) compared to original splicing reporter (panels III to VI), taken at the early adult stage. Schematics showing the gene constructs used for the alternative splicing reporter strain. “D” and “v” indicate dorsal and ventral sides, respectively. (TIF) [file pgen.1011109.s006.tif]

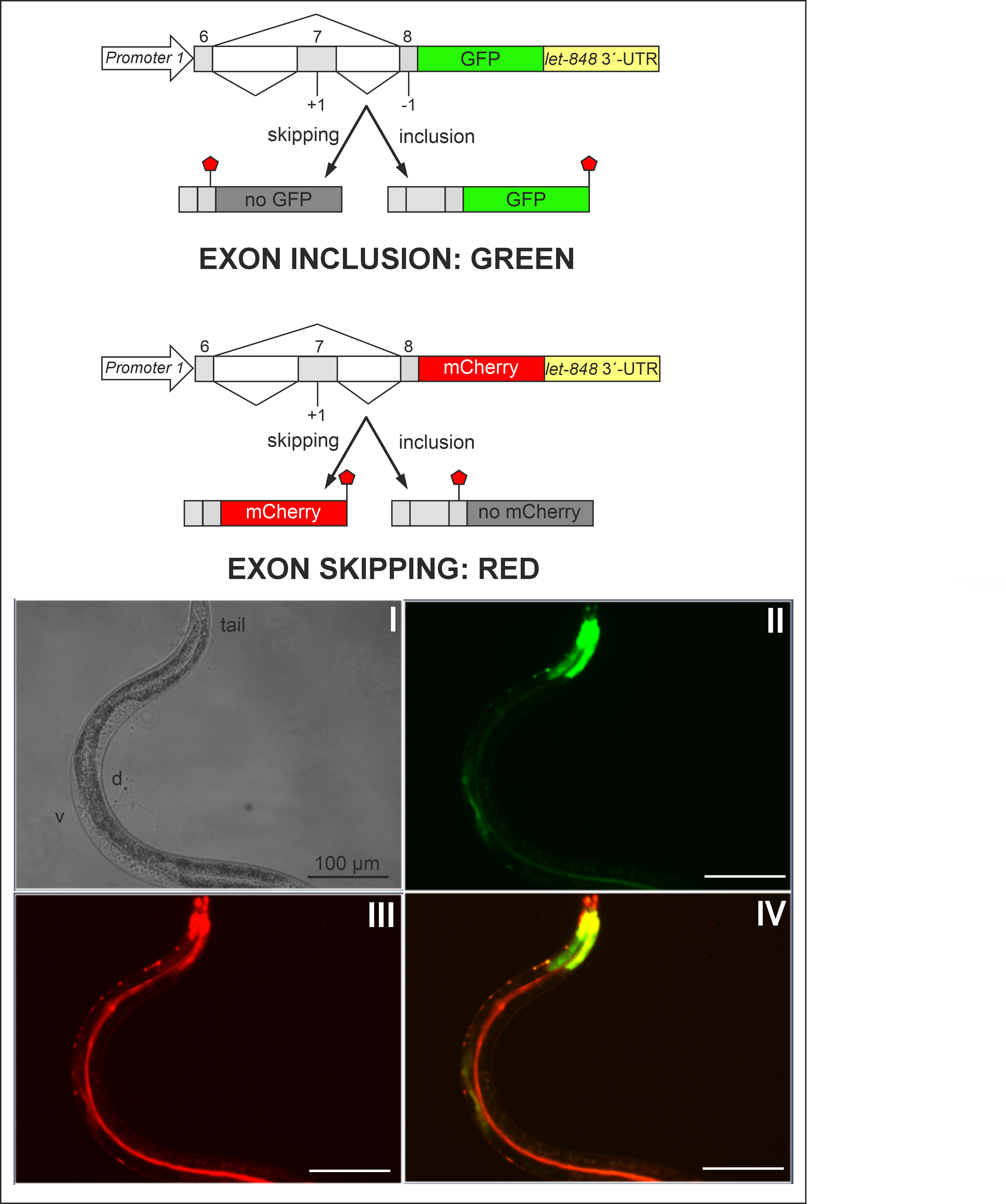

Supplement: S7 Fig — DIC and fluorescence images of alternative splicing reporter animals for which the unc-54 3′-UTR was replaced with the let-848 3′-UTR, taken at the L3 stage. Schematics showing the gene constructs used for the alternative splicing reporter strain. “D” and “v” indicate dorsal and ventral sides, respectively. (TIF) [file pgen.1011109.s007.tif]

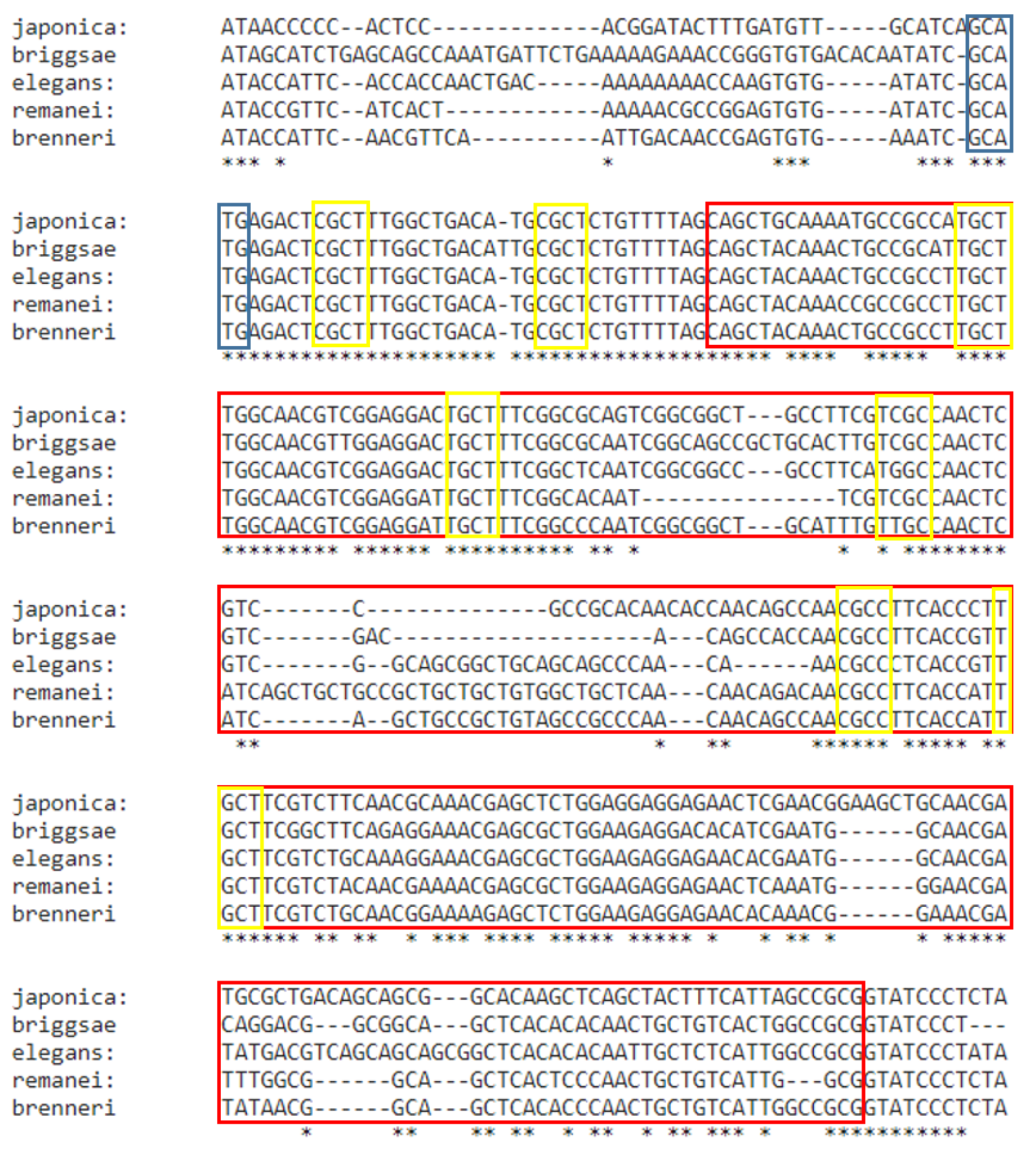

Supplement: S8 Fig — Multiple sequence alignment of the mbl-1 genes from five representative species of the Caenorhabditis genus. Exon 7 in red, putative conserved MBL-1 binding sites (YGCY) in yellow, and putative conserved FOX-1 binding site (GCAUG) in blue. Asterisks indicate nucleotides with 100% sequence conservation. (TIF) [file pgen.1011109.s008.tif]

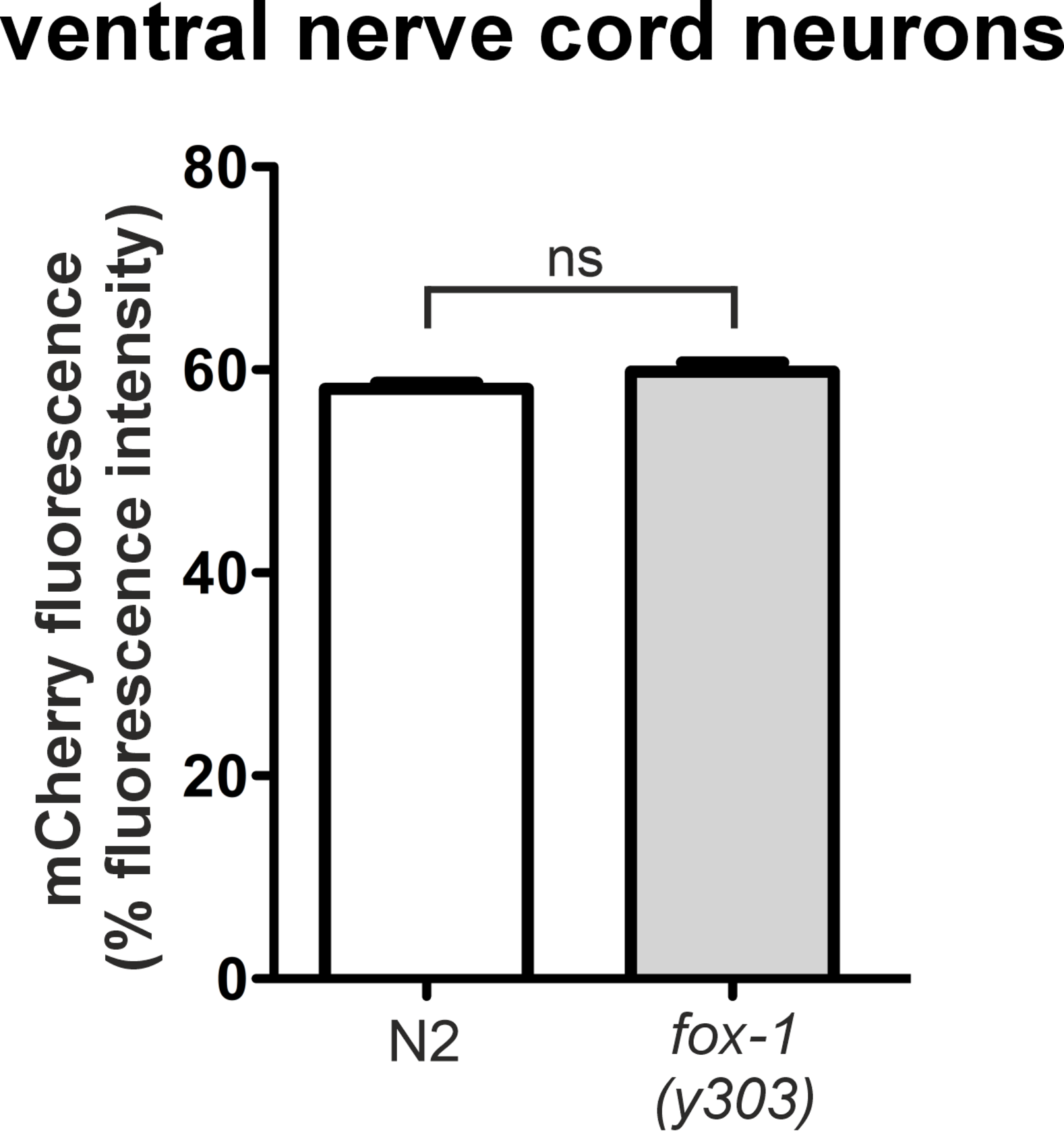

Supplement: S9 Fig — Quantification of relative mCherry fluorescence intensity in the VNC neurons from indicated animals expressing the fluorescence alternative splicing reporter minigenes at L4 stage. For each strain, a total population of 30 worms was analyzed, grown on two different plates (15 animals per plate). An unpaired two-sample Student’s t-test was performed (ns: no significance). Error bars indicate SEM. (TIF) [file pgen.1011109.s009.tif]

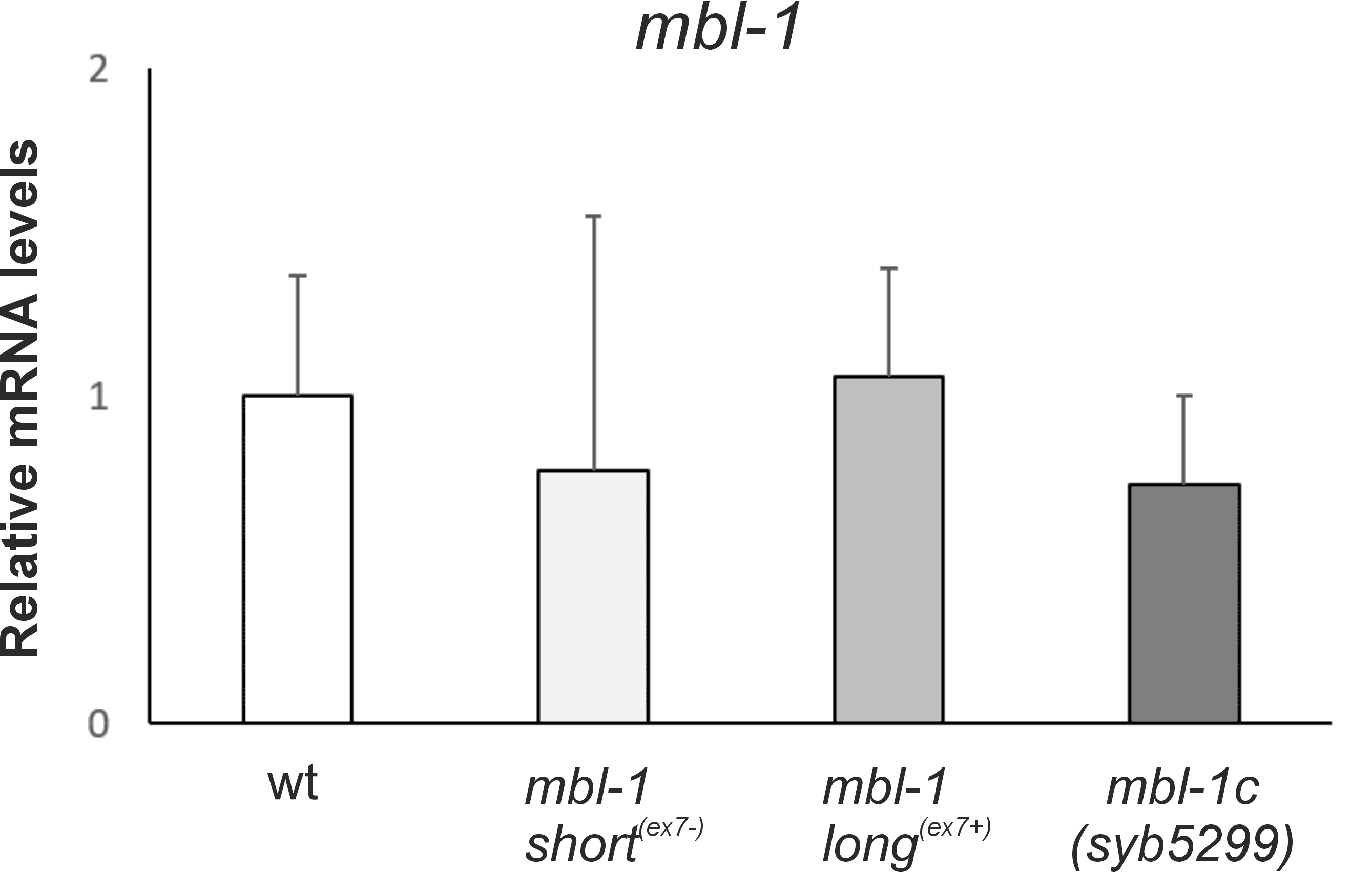

Supplement: S10 Fig — Transcripts levels of mbl-1 from L4 animals were measured by qPCR. Experiments were done with three biological repeats (each with three technical replicates) with RNA collected from animals grown on different plates. One-way ANOVA with Dunnett’s multiple comparisons test was performed and no significant differences were found. Error bars indicate SEM. (TIF) [file pgen.1011109.s010.tif]

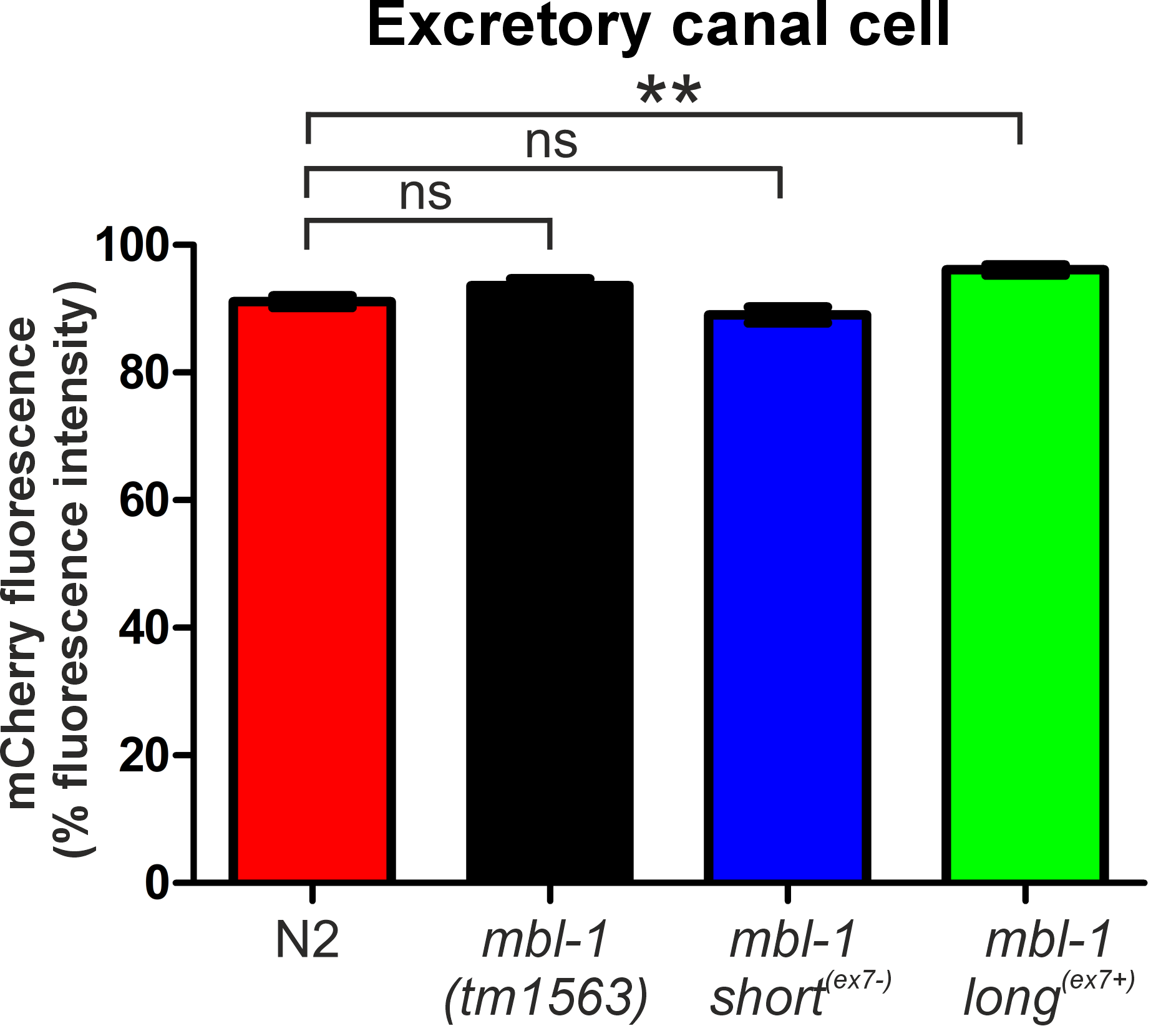

Supplement: S11 Fig — Quantification of relative mCherry fluorescence intensity in the excretory canal cells from animals at the L4 stage expressing the fluorescence alternative splicing reporter minigenes. For each strain, a total population of 30 worms was analyzed, grown on two different plates (15 animals per plate). One-way ANOVA with Dunnett´s multiple comparison test was performed (*** P < 0.001; ns: no significance). Error bars indicate SEM. (TIF) [file pgen.1011109.s011.tif]

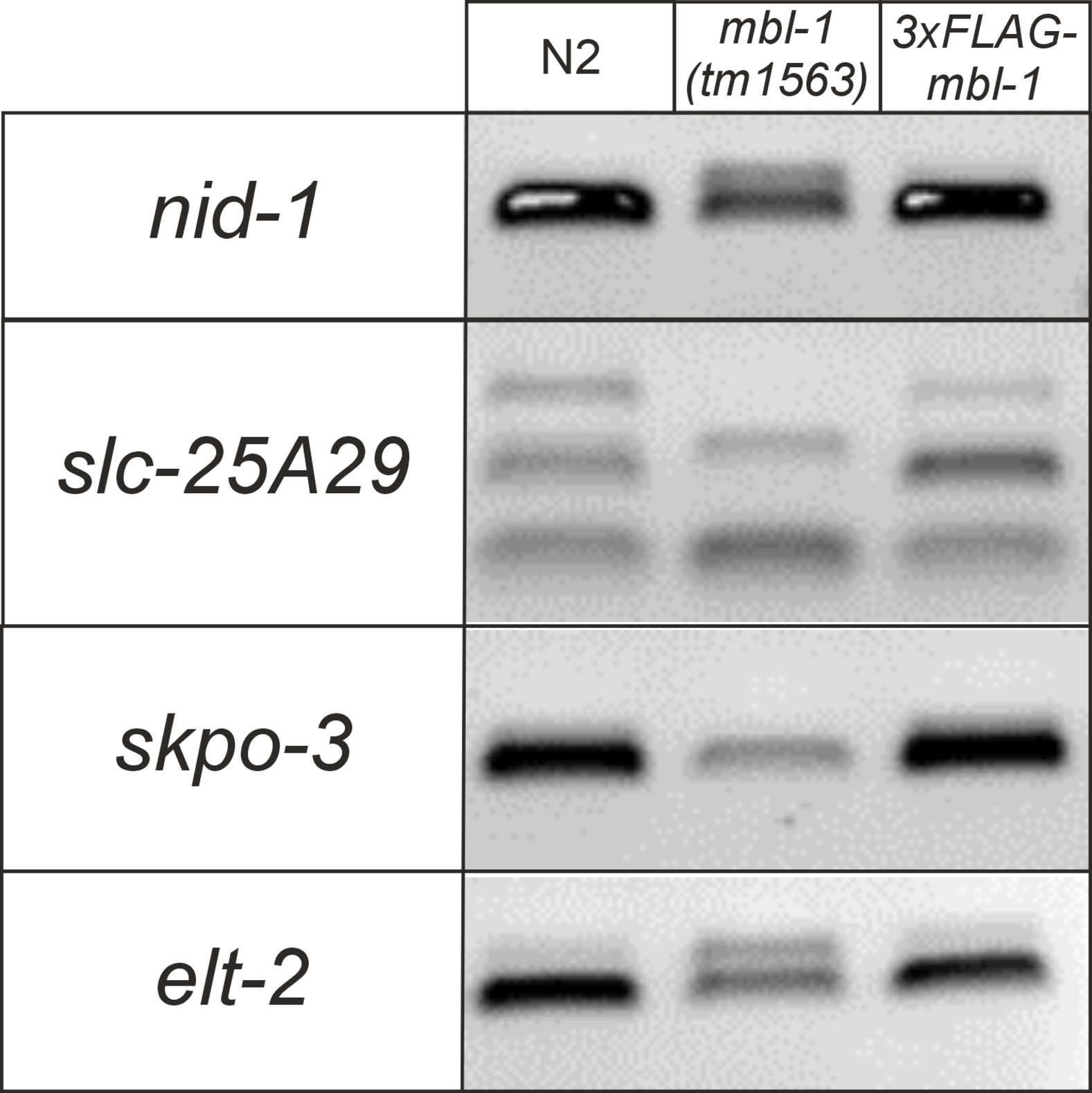

Supplement: S12 Fig — RT-PCR analysis of mCherry::MBL-1 splicing functionality at the L4 stage. (TIF) [file pgen.1011109.s012.tif]

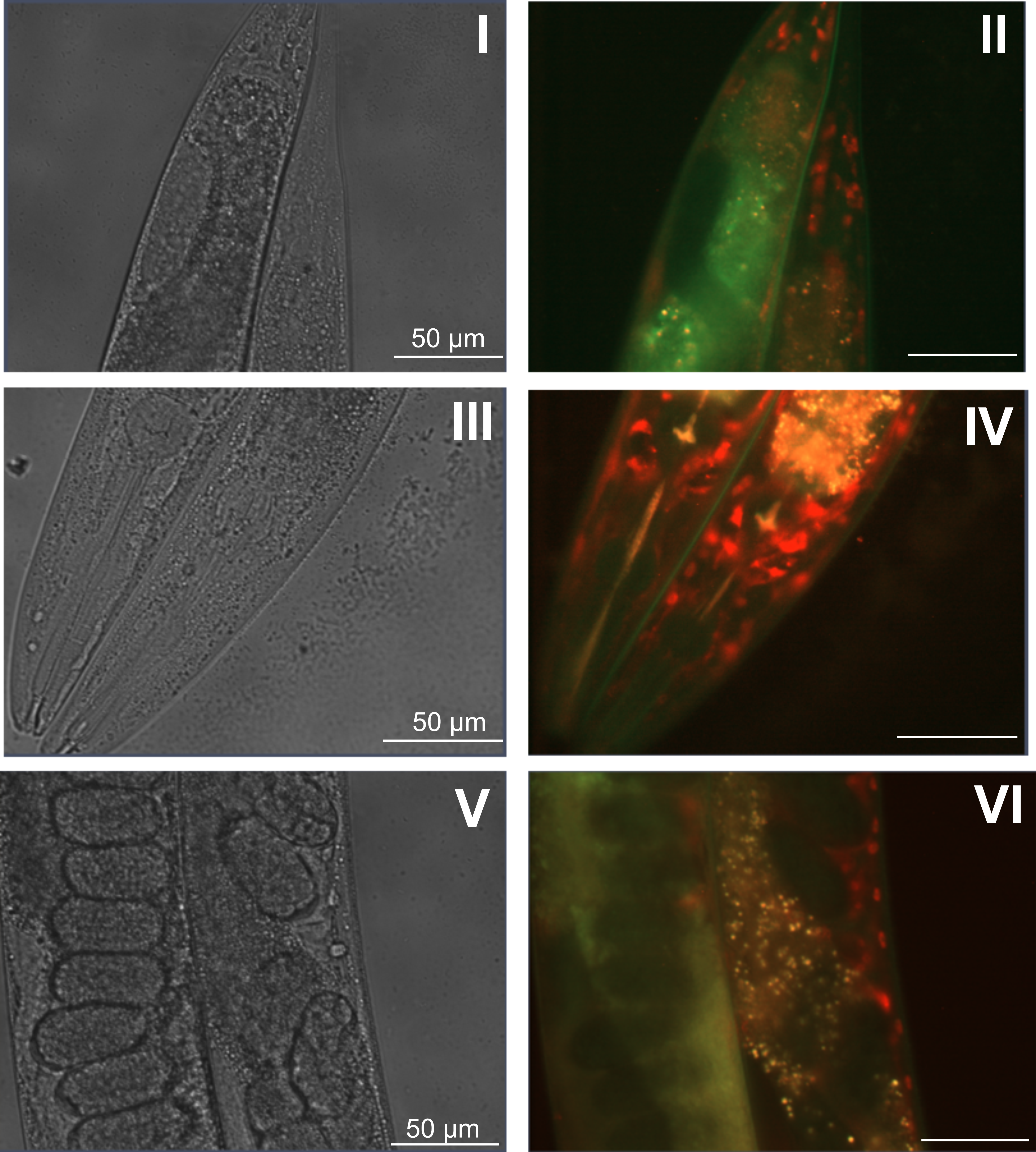

Supplement: S13 Fig — Representative DIC and fluorescence images of adult 3XFLAG::MBL-1::mCherry animals. Images represent tail (panels I and II), head (III and IV) and vulvar region (V and VI). Green signals are the result of autofluorescence. (TIF) [file pgen.1011109.s013.tif]

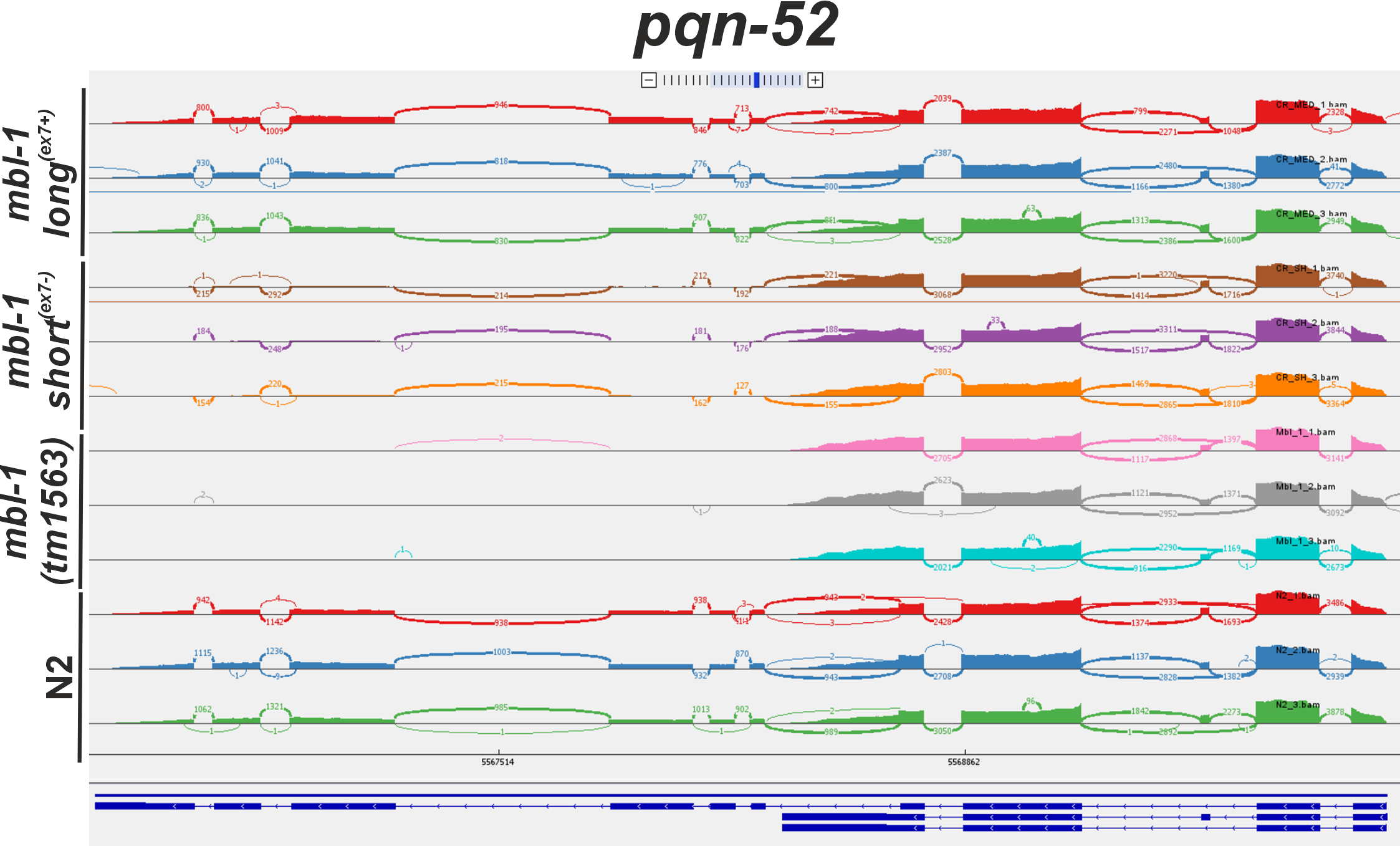

Supplement: S14 Fig — Sashimi plots of the pqn-52 gene from RNA-seq data derived from animals at the L4 stage showing regulation of alternative polyadenylation by MBL-1. Gene model with exons in blue boxes at the bottom. Numbers represent exon spanning reads. (TIF) [file pgen.1011109.s014.tif]
