# Supplementary material for: The Muscleblind-like protein MBL-1 regulates microRNA expression in Caenorhabditis elegans through an evolutionarily conserved autoregulatory mechanism
Source: PLoS Genet. 2023 Dec 22;19(12):e1011109. doi: 10.1371/journal.pgen.1011109 (PMC10773944; doi:10.1371/journal.pgen.1011109)
Supplement: S1 Appendix — (DOCX) [file pgen.1011109.s021.docx]

**S1 Appendix. List of strains used and created within this study.**

*Strains obtained from CGC or NRBP:*

**CZ10175**: z*dIs5[Pmec-4::GFP + lin-15(+)]*

**TY2685:** *fox-1(y303)*

**VC446**: *alg-1(gk214)*

*mbl-1(tm1563)*

*Strains generated within this study:*

**GAR153:** *iceIs50[Pmbl-1(1)::mbl-1-short::mCherry::unc-54 3*′*-UTR + Pmbl-1(2)::mbl-1-short::mCherry::unc-54 3*′*-UTR]*

**GAR154:** *iceIs51[Pmbl-1(1)::mbl-1-long::mCherry::unc-54 3*′*-UTR + Pmbl-1(2)::mbl-1-long::mCherry::unc-54 3*′*-UTR]*

**GAR184:** *iceIs52[Pmbl-1(1)::mbl-1-ex7(+1)::mCherry::unc-54 3*′*-UTR + Pmbl-1(1)::mbl-1-ex7(-1)::GFP::unc-54 3*′*-UTR]*

**GAR185:** *iceEx53[Pmbl-1(1)::mbl-1-ex7(+1)::mCherry::let-848 3*′*-UTR + Pmbl-1(1)::mbl-1-ex7(-1)::GFP::let-848 3*′*-UTR]*

**GAR186:** *iceEx54[Pmbl-1(1)::mbl-1-ex7(-1)::mCherry::unc-54 3*′*-UTR + Pmbl-1(1)::mbl-1-ex7(+1)::GFP::unc-54 3*′*-UTR]*

**GAR187:** *iceEx55[Pmbl-1(2)::mbl-1-ex7(+1)::mCherry::unc-54 3***′***-UTR + Pmbl-1(2)::mbl-1-ex7(-1)::GFP::unc-54 3*′*-UTR]*

**GAR188:** *zdIs5[Pmec-4::GFP + lin-15(+)]; mbl-1(syb4318)*

**GAR189:** *iceIs52; mbl-1(tm1563)*

**GAR190:** *iceIs52; mbl-1(syb4318)*

**GAR 191:** *iceIs52; fox-1(y303)*

**GAR193:** *iceIs50; mbl-1(tm1563)*

**GAR194:** *iceIs51; mbl-1(tm1563)*

**GAR195:** *iceIs50; iceis51; mbl-1(tm1563)*

**GAR196:** *iceIs52; mbl-1(syb4345)*

**GAR197:** *zdIs5[Pmec-4::GFP + lin-15(+)]; mbl-1(syb4345)*

**GAR198:** *zdIs5[Pmec-4::GFP + lin-15(+)]; mbl-1(tm1563)*

*Strains generated through CRISPR/Cas9 genomic editing:*

**PHX4318:** *mbl-1(syb4318)*

**PHX4345**: *mbl-1(syb4345)*

**PHX5299**: *mbl-1c(syb5299)*
