# Supplementary material for: The Muscleblind-like protein MBL-1 regulates microRNA expression in Caenorhabditis elegans through an evolutionarily conserved autoregulatory mechanism
Source: PLoS Genet. 2023 Dec 22;19(12):e1011109. doi: 10.1371/journal.pgen.1011109 (PMC10773944; doi:10.1371/journal.pgen.1011109)
Supplement: S2 Appendix — (DOCX) [file pgen.1011109.s022.docx]

-:deletion site

**Strain name and genotype:**

PHX4318 *mbl-1 (syb4318); mbl-1 short^(ex7-)^*

**PCR and sequencing primers:**

SUG01-ko-s: AAAGCTTCACCTCTACTATG

SUG01-ko-a: TTTCTACCTAATGGCTCAAC

>SUG01-syb4318(-825bp)

aaagcttcacctctactatgcttgtcgtggcccctgttattgagttttagattttatggataaaattagttccaaaaataaaagtgaataacaatttggaaactcgaaaaaatcgatcaaactcggaaagttaaatgcaaaatttttcctgaacacttttcaaatcaattttcacgtaatgtagattttgttactcaaacctgaaatcagtgaaatagctttttaagacttttaagcctgagaaattaggcttacgcttatgcttgaaaatactatacataaaaactgagcttaagcacttgacaatttacggatttgtgtttacttggcactgtgtcttgtaagtaaaagttttgcattgaactttttaccgatcagtttctgaggtttgaggaacaattgcatagctacata-atgtgtatcaatggagtgtttgtaagcatgtaaatgtatggggttaggtgcaatttttcaattgatttttcgtacttcaacttttcaaattctaacggagactacatatatacatttttctaaagcacttagattgtagtgaagtttacgattttggttgggttttgggggatgaatatgagttgctgaaaatttaaatattgcaaatgaaatgctcaattttataagctgttgttttagcattaaagatacagtattgcaagttaaatagggataaaacgagcagataccattgtgaattattttgaaatctttctgaattgctttattagattttgcaacaaaaaaaattttaagattttctatataatttcgtgctaaaatgtttgaaatgtatttttacctgaatttttttttgagttttttgcataatttgaaacaaacaacagagttcccgttttggctactttatgtcctatttaatgttttgatttattaataaactggaatatttgcttctaaagttgagccattaggtagaaa

**>** Wild type

aaagcttcacctctactatgcttgtcgtggcccctgttattgagttttagattttatggataaaattagttccaaaaataaaagtgaataacaatttggaaactcgaaaaaatcgatcaaactcggaaagttaaatgcaaaatttttcctgaacacttttcaaatcaattttcacgtaatgtagattttgttactcaaacctgaaatcagtgaaatagctttttaagacttttaagcctgagaaattaggcttacgcttatgcttgaaaatactatacataaaaactgagcttaagcacttgacaatttacggatttgtgtttacttggcactgtgtcttgtaagtaaaagttttgcattgaactttttaccgatcagtttctgaggtttgaggaacaattgcatagctacataccaggttgattttgagcttgatggaacgaattttatattgggttttaacaatgattgttgtgttattttctattagaacaaccctatcccctatttttgattttcgtaatacaaatattgatttctaatttgaatatttattttgggtagccttttgaattggaatgtagtaaattaagttttccgagcttttcgagGTGCTCGACTCAAATCAAGAATACCATTCACCACCAACTGACAAAAAAAACCAAgtgtgatatcgcatgagactcgctttggctgacatgcgctctgttttagCAGCTACAAACTGCCGCCTTGCTTGGCAACGTCGGAGGACTGCTTTCGGCTCAATCGGCGGCCGCCTTCATGGCCAACTCGTCGGCAGCGGCTGCAGCAGCCCAACAAACGCCCTCACCGTTGCTTCGTCTGCAAAGGAAACGAGCGCTGGAAGAGGAGAACACGAATGGCAACGATATGACGTCAGCAGCAGCGGCTCACACACAATTGCTCTCATTGGCCGCGgtatccctatatcttgttttttttttgaattctgacaacaaaactttggtttttatccttcttcaaaatttttggttttatatctcttctctcttatctttcctagaaacgctgctgttaactttaactttaataaccaaaaactttgaacgattctggtactggccatgattacggttaataactgcttgcttgtcttgtttctcttgcatctcgaattcaaattttagaatttgtgtaaaagttcctaagtttttacccccgaatgtttgcaagtaccaaaatcgttagatgtgcctaatgtgtatcaatggagtgtttgtaagcatgtaaatgtatggggttaggtgcaatttttcaattgatttttcgtacttcaacttttcaaattctaacggagactacatatatacatttttctaaagcacttagattgtagtgaagtttacgattttggttgggttttgggggatgaatatgagttgctgaaaatttaaatattgcaaatgaaatgctcaattttataagctgttgttttagcattaaagatacagtattgcaagttaaatagggataaaacgagcagataccattgtgaattattttgaaatctttctgaattgctttattagattttgcaacaaaaaaaattttaagattttctatataatttcgtgctaaaatgtttgaaatgtatttttacctgaatttttttttgagttttttgcataatttgaaacaaacaacagagttcccgttttggctactttatgtcctatttaatgttttgatttattaataaactggaatatttgcttctaaagttgagccattaggtagaaa

**Strain name and genotype:**

PHX4345 *mbl-1(syb4345)*; *mbl-1 long^(ex7+)^*

**PCR and sequencing primers:**

SUG02-ko-s:TTGAACCTAATCTGAGCACA

SUG02-ko-a:CCATTGATACACATTAGGCA

**>SUG02-syb4345 (-1266bp)**

ttgaacctaatctgagcacatgttccattttttaatttttgtttgattatgggttgtgagcaataaaatggtaccaataaatatagtatttcctctatcaattcttactttttttgggttttggcagctttctatatgtagttcgatcgaaaattgtaagctaacaatttttctttaagtaaagcatttaaaggaattcttaaagttacaaaaaaattcagagaccttaaatttacaatttttctcaaaataatgttgtcaaatattctcactaggtaaactattcaagttcaaaatattttccgttccagCAACAACAAGCGGCCGCAGTAAACCTGATTCCCAACACACCAATTTACCCACCCTACTACAACGGCATGATGTATCCACAAGTACTTCAAGACCCTTATACAGCAGCGGCAGTGAATCAG-CAGCTACAAACT**GCa**GCCTTGCTTGGCAACGTCGGAGGACTGCTTTCGGCTCAATCGGCGGCCGCCTTCATGGCCAACTCGTCGGCAGCGGCTGCAGCAGCCCAACAAACGCCCTCACCGTTGCTTCGTCTGCAAAGGAAACGAGCGCTGGAAGAGGAGAACACGAATGGCAACGATATGACGTCAGCAGCAGCGGCTCACACACAATTGCTCTCATTGGCCGCGgtatccctatatcttgttttttttttgaattctgacaacaaaactttggtttttatccttcttcaaaatttttggttttatatctcttctctcttatctttcctagaaacgctgctgttaactttaactttaataaccaaaaactttgaacgattctggtactggccatgattacggttaataactgcttgcttgtcttgtttctcttgcatctcgaattcaaattttagaatttgtgtaaaagttcctaagtttttacccccgaatgtttgcaagtaccaaaatcgttagatgtgcctaatgtgtatcaatgg

**>wild type**

ttgaacctaatctgagcacatgttccattttttaatttttgtttgattatgggttgtgagcaataaaatggtaccaataaatatagtatttcctctatcaattcttactttttttgggttttggcagctttctatatgtagttcgatcgaaaattgtaagctaacaatttttctttaagtaaagcatttaaaggaattcttaaagttacaaaaaaattcagagaccttaaatttacaatttttctcaaaataatgttgtcaaatattctcactaggtaaactattcaagttcaaaatattttccgttccagCAACAACAAGCGGCCGCAGTAAACCTGATTCCCAACACACCAATTTACCCACCCTACTACAACGGCATGATGTATCCACAAGTACTTCAAGACCCTTATACAGCAGCGGCAGTGAATCAGgtacggtaaaaacagcttgaaccttcttccctcacaatccctctttgcaccttgcatcattgtattgctattcgcaccaccgattttctacggaaaaaaaaattttgttttgatttggaggaggaggatcaaggaatcagaattctcaccttgcatgggtattttgattgattttgtgatgaagaatttaaatttaaaataatttcaataatggactacccaaacattcaattttgtttccgaaaattcaaaaatccgacccacctctcttcctgccttttataatttttcggttgcgaacgctcgctcgctgtgctcctattcttaccacgcacaaacaaacaaacaaacaaacacatcttctcaaagcacgtctcatgttagttttggtcctattttcaaacatatttgtttaaatcaagcaaaaaaaagaaacaacagaaagcggtgaatgttgcacaaggtgtctttggaatggatgttattcatttgatttgaatctttcaccacattgcccggcttttgtaaaaatttcagaaaataaattctcaaaaagcttcacctctactatgcttgtcgtggcccctgttattgagttttagattttatggataaaattagttccaaaaataaaagtgaataacaatttggaaactcgaaaaaatcgatcaaactcggaaagttaaatgcaaaatttttcctgaacacttttcaaatcaattttcacgtaatgtagattttgttactcaaacctgaaatcagtgaaatagctttttaagacttttaagcctgagaaattaggcttacgcttatgcttgaaaatactatacataaaaactgagcttaagcacttgacaatttacggatttgtgtttacttggcactgtgtcttgtaagtaaaagttttgcattgaactttttaccgatcagtttctgaggtttgaggaacaattgcatagctacataccaggttgattttgagcttgatggaacgaattttatattgggttttaacaatgattgttgtgttattttctattagaacaaccctatcccctatttttgattttcgtaatacaaatattgatttctaatttgaatatttattttgggtagccttttgaattggaatgtagtaaattaagttttccgagcttttcgagGTGCTCGACTCAAATCAAGAATACCATTCACCACCAACTGACAAAAAAAACCAAgtgtgatatcgcatgagactcgctttggctgacatgcgctctgttttagCAGCTACAAACT**GCC**GCCTTGCTTGGCAACGTCGGAGGACTGCTTTCGGCTCAATCGGCGGCCGCCTTCATGGCCAACTCGTCGGCAGCGGCTGCAGCAGCCCAACAAACGCCCTCACCGTTGCTTCGTCTGCAAAGGAAACGAGCGCTGGAAGAGGAGAACACGAATGGCAACGATATGACGTCAGCAGCAGCGGCTCACACACAATTGCTCTCATTGGCCGCGgtatccctatatcttgttttttttttgaattctgacaacaaaactttggtttttatccttcttcaaaatttttggttttatatctcttctctcttatctttcctagaaacgctgctgttaactttaactttaataaccaaaaactttgaacgattctggtactggccatgattacggttaataactgcttgcttgtcttgtttctcttgcatctcgaattcaaattttagaatttgtgtaaaagttcctaagtttttacccccgaatgtttgcaagtaccaaaatcgttagatgtgcctaatgtgtatcaatgg

**Strain name and genotype:**

PHX5299 *mbl-lc(syb5299)*; *3XFLAG::mCherry::MBL-1*

Synonymous mutation is labelled in blue**.**

**PCR and sequencing primers:**

SUG03-seq-s:caacccaccaaacacttc

SUG03-seq-a:cacacgccaaccaatccc

**>**SUG03**-**syb5299

caacccaccaaacacttctacgtcttcctattcttattcaaccatctttttttctcgctatttgtttttttatttgttcggtctcttttcctcatccactgaaaatttatgaacctaatctctaacacccatttttgttcaatttcttcatatcttcccgttcgattcggttattgaataggtagaatcttcaaaaaactggttttcaatcaggccatgttgtgttcttttatcactgttctaattaaccattaatatgacgttggaaagctcaaacatttagctgtcagagcattcttaaagttaccgtaataaactgactcgagatgatttgaatctcatcttgatgctactgtaattttaaaatgggcactgtagctattttggtcttcccgatagcatttaccttacaagttcttcgaccatttttgggaagaaagaaaaaatgaaataagggcgcctagaaagaaaaaaaaactttcgtactattgtgactcaacagttgttgcctctttcgttccccccgtcgcctccgccccaagtttggaggcctaacaaaaaataattctcattgccaatctttttcctacttttcagaATGGATTATAAAGATCATGATGGAGATTATAAAGATCATGATATTGATTATAAAGATGATGATGATAAAGGAGGAGGAGGAGGAGGAATGGTCTCAAAGGGTGAAGAAGATAACATGGCAATTATTAAAGAGTTTATGCGTTTCAAGGTGCATATGGAGGGATCTGTCAATGGGCATGAGTTTGAAATTGAAGGTGAAGGAGAAGGCCGACCATATGAGGGAACACAAACCGCAAAACTAAAGgtaagtttaaacatatatatactaactaaccctgattatttaaattttcagGTAACTAAAGGCGGACCATTACCATTCGCCTGGGACATCCTCTCTCCACAGTTCATGTATGGAAGTAAAGCTTATGTTAAACATCCGGCAGATATACCAGATTATTTGAAACTTTCATTCCCGGAGGGTTTTAAGTGGGAACGCGTAATGAATTTTGAAGACGGAGGAGTTGTTACAGTGACGCAAGACTCAAGgtaagtttaaacagttcggtactaactaaccatacatatttaaattttcagCCTCCAAGATGGAGAATTTATTTATAAAGTCAAACTTCGAGGAACGAATTTCCCCTCGGATGGACCTGTTATGCAGAAGAAGACTATGGGATGGGAAGCTTCAAGTGAAAGAATGTACCCTGAAGACGGTGCTCTTAAGGGAGAGATTAAACAACGTCTTAAATTGAAAGATGGAGGACATTACGATGCTGAGgtaagtttaaacatgattttactaactaactaatctgatttaaattttcagGTGAAGACAACTTACAAAGCCAAAAAACCAGTTCAGCTGCCAGGAGCGTACAATGTTAATATTAAACTGGATATCACCTCCCACAACGAGGATTACACTATCGTTGAGCAATATGAAAGAGCTGAAGGGCGGCACTCGACAGGTGGCATGGATGAATTGTATAAGGGAGGAGGAGGAGGAGGAATGTTCGACGAAAACAGT**AAcGCt**GCTGGAACGACGCCT**GTgGCa**TCATCGCTTGCCGCCACGCCTAATGCCAATCTCGTGTCACAAGTGTTCAATGTGAAAGACAGCCGATGGTTACAGgtatgtctatatggaaacttttgattttggaaactacggagaccaaaattatataaaaccgtagtcaccttccttaacgcgtacactcttaccaaataacaaaatctttatttcggcatcctatccaagaaaaaaatcaataatggtttttcattttcccatcgctataatttccacgaagcgttcacatagtttaagaggtaattcatatactttgggacagcttgctcttcttccaatttcgcatcgggttttcccgcgcacacgtaataggagacgttattaagatgcgctcatgtggcgtttgacgccacgggaaaacagaagacgtttgagcgactctaaggctcttcgggattggttggcgtgtg
