# Supplementary material for: The Muscleblind-like protein MBL-1 regulates microRNA expression in Caenorhabditis elegans through an evolutionarily conserved autoregulatory mechanism
Source: PLoS Genet. 2023 Dec 22;19(12):e1011109. doi: 10.1371/journal.pgen.1011109 (PMC10773944; doi:10.1371/journal.pgen.1011109)
Supplement: S3 Appendix — (DOCX) [file pgen.1011109.s023.docx]

| **Genotype** | **No. of subjects** | **Mean Lifespan + SE (days)** | P-value vs. N2 | P-value vs. *mbl-1(tm1563)* | P-value vs. *mbl-1 short* | P-value vs. *mbl-1 long* |
| --- | --- | --- | --- | --- | --- | --- |
| N2 | 216 | 21.53 + 0.33 | 1 | < 10^-3^ | 0.95 | < 10^-3^ |
| *mbl-1(tm1563)* | 220 | 14.45 + 0.18 | < 10^-3^ | 1 | < 10^-3^ | 0.001 |
| *mbl-1 short^(ex7-)^* | 255 | 21.58 + 0.24 | 0.95 | < 10^-3^ | 1 | < 10^-3^ |
| *mbl-1 long^(ex7+)^* | 235 | 15.38 + 0.22 | < 10^-3^ | 0.001 | < 10^-3^ | 1 |

**Lifespan data**

**FIG 3B**

**Lifespan summary**

**Individual lifespans**

| **Genotype** | **No. of subjects** | **Mean Lifespan + SE (days)** | P-value vs. N2 | P-value vs. *mbl-1(tm1563)* | P-value vs. *mbl-1 short* | P-value vs. *mbl-1 long* |
| --- | --- | --- | --- | --- | --- | --- |
| N2 | 60 | 21.72 + 0.55 | 1 | < 10^-3^ | 1 | < 10^-3^ |
| *mbl-1(tm1563)* | 75 | 14.58 + 0.28 | < 10^-3^ | 1 | < 10^-3^ | 1 |
| *mbl-1 short^(ex7-)^* | 75 | 22.16 + 0.46 | 1 | < 10^-3^ | 1 | < 10^-3^ |
| *mbl-1 long^(ex7+)^* | 55 | 15.15 + 0.54 | < 10^-3^ | 1 | < 10^-3^ | 1 |

| **Genotype** | **No. of subjects** | **Mean Lifespan + SE (days)** | P-value vs. N2 | P-value vs. *mbl-1(tm1563)* | P-value vs. *mbl-1 short* | P-value vs. *mbl-1 long* |
| --- | --- | --- | --- | --- | --- | --- |
| N2 | 88 | 20.18 + 0.51 | 1 | < 10^-3^ | 1 | < 10^-3^ |
| *mbl-1(tm1563)* | 90 | 13.74 + 0.29 | < 10^-3^ | 1 | < 10^-3^ | 0.0116 |
| *mbl-1 short^(ex7-)^* | 90 | 20.90 + 0.36 | 1 | < 10^-3^ | 1 | < 10^-3^ |
| *mbl-1 long^(ex7+)^* | 90 | 14.99 + 0.32 | < 10^-3^ | 0.0116 | < 10^-3^ | 1 |

| **Genotype** | **No. of subjects** | **Mean Lifespan + SE (days)** | P-value vs. N2 | P-value vs. *mbl-1(tm1563)* | P-value vs. *mbl-1 short* | P-value vs. *mbl-1 long* |
| --- | --- | --- | --- | --- | --- | --- |
| N2 | 68 | 22.14 + 0.45 | 1 | < 10^-3^ | 0.05 | < 10^-3^ |
| *mbl-1(tm1563)* | 55 | 15.23 + 0.31 | < 10^-3^ | 1 | < 10^-3^ | 0.31 |
| *mbl-1 short^(ex7-)^* | 90 | 21.59 + 0.40 | 0.05 | < 10^-3^ | 1 | < 10^-3^ |
| *mbl-1 long^(ex7+)^* | 90 | 16.00 + 0.34 | < 10^-3^ | 0.31 | < 10^-3^ | 1 |

**FIG 7D**

| **Genotype + treatment** | **No. of subjects** | **Mean Lifespan + SE (days)** | **Variation compared to EV control** |
| --- | --- | --- | --- |
| N2, EV | 68 | 22.14 + 0.45 | - |
| N2, *alg-1* | 60 | 14.86 + 0.14 | - 32.9 % |
| *mbl-1 short^(ex7-)^*, EV | 90 | 21.59 + 0.40 | - |
| *mbl-1 short^(ex7-)^*, *alg-1* | 90 | 14.92 + 0.23 | -30.9 % |

| **Genotype + treatment** | **No. of subjects** | **Mean Lifespan + SE (days)** | **Variation compared to EV control** |
| --- | --- | --- | --- |
| *mbl-1(tm1563)*, EV | 55 | 15.23 + 0.31 | - |
| *mbl-1(tm1563)*, *alg-1* | 90 | 12.81 + 0.21 | -15.9 % |
| *mbl-1 long^(ex7+)^*, EV | 90 | 16.00 + 0.34 | - |
| *mbl-1 long^(ex7+)^*, *alg-1* | 90 | 13.26 + 0.21 | -17.1 % |
