# Supplementary material for: The Muscleblind-like protein MBL-1 regulates microRNA expression in Caenorhabditis elegans through an evolutionarily conserved autoregulatory mechanism
Source: PLoS Genet. 2023 Dec 22;19(12):e1011109. doi: 10.1371/journal.pgen.1011109 (PMC10773944; doi:10.1371/journal.pgen.1011109)
Supplement: S4 Appendix — (DOCX) [file pgen.1011109.s024.docx]

**Oligonucleotide sequences used for RT-PCR splicing analysis in this study (Fig 4D, S12 Fig).**

| Gene | Forward (5’-3’) | Reverse (5’-3’) |
| --- | --- | --- |
| *mbl-1* | ACCTGATTCCCAACACACCA | TTGCTCCGTTCTTGTCGAGA |
| *blmp-1* | ATCAATGTCAGGGTCGGGTT | TGCTGCTGTTGAGATGTTGG |
| *unc-43* | GACCACCATCGACAATCAAGG | CAACAACGTCTGAGTCACCC |
| *unc-36* | AGCACTTCCGTTAGCCTTCA | CGATTGCTTCATCTCCAACA |
| *nid-1* | AAGCTGCGCTTATCACCACT | CGTTCAAATATGGCCATTCC  GACAGACGCATCGGAATGTA |
| *slc-25A29* | CATCAAACGCAGCAACATTC | TGATTGTTTGTTGCCCAGAA  GACAACGTCAATTCCTCGAA |
| *skpo-3* | TCCACCACCAGTTACTCGTC | GGAGTTGGTCGAATCGTTGG |
| *elt-2* | CCAGACTGGCCTAAGACCTG | CAACTTTGGAAGAGCTGAGCA |

**Oligonucleotide sequences used for amplification of *mbl-1* transcripts (Fig 1A).**

| Name | Forward (5’-3’) | Reverse (5’-3’) |
| --- | --- | --- |
| *primer A* | GAGAAAGGTGCTATCCAGGC |  |
| *primer B* | GCCAATCTTTTTCCTACTTTTCAGA |  |
| *primer C* |  | GGGTAGGTGGTGAGAAAAATTA |
| *primer D* |  | TCTAGTAGCGAGGCGGTAGC |

**Oligonucleotide sequences used for qRT-PCR in this study (Fig 1E, 5D, S10 Fig)**

| Gene | Forward (5’-3’) | Reverse (5’-3’) |
| --- | --- | --- |
| *mbl-1 short* | GCAGTGAATCAGGGAGCTG | GCTGGCACGTATCCTTGAAG |
| *mbl-1 long* | CACACAATTGCTCTCATTGG | GCTGGCACGTATCCTTGAAG |
| *mbl-1 total* | GCTGTACCAATGAAGCGACC | GCTGGCACGTATCCTTGAAG |
| *rps-23* | CTCACATTGGAACTCGCTGG | TCTTGATGAGCTGGACACGA |
| *mec-7* | CGTCTACTATAATGAGGCCGGA | CCCTCGGTGTAGTGTCCTTT |
| *mec-12* | CTTCTGACAAGTCCCTCGGA | AGTGATGAGTTGTTCCGGGT |
| *mec-17* | TGGGATTCGCGAAAGTAGGA | TGATAAGGTTCAGTGTGTTCCTG |
| *ben-1* | GGAACTGGATCTGGAATGGGA | GCCTCGTTGTCAATGCAGAA |

**Oligonucleotide sequences used for genotyping of *mbl-1* transcripts**

| Gene | Forward (5’-3’) | Reverse (5’-3’) | Reverse (5’-3’) |
| --- | --- | --- | --- |
| *mbl-1* | ACCTGATTCCCAACACACCA |  |  |
| *mbl-1* | TTTTTACCGATCAGTTTCTGAGG |  |  |
| *mbl-1* |  | CCTTTGCAGACGAAGCAAC |  |
| *mbl-1* |  | AGGTGCAAAGAGGGATTGTG |  |
| *mbl-1* |  | CAACCAAAATCGTAAACTTCACT |  |
| *mbl-1* |  | TCAAAAATAGGGGATAGGGTTG |  |
