# Supplementary material for: The Muscleblind-like protein MBL-1 regulates microRNA expression in Caenorhabditis elegans through an evolutionarily conserved autoregulatory mechanism
Source: PLoS Genet. 2023 Dec 22;19(12):e1011109. doi: 10.1371/journal.pgen.1011109 (PMC10773944; doi:10.1371/journal.pgen.1011109)
Supplement: Attachment — Submitted filename: Response_to_reviewers_Jens_Verbeeren.pdf [file pgen.1011109.s028.pdf]

Institute of Biotechnology
HiLIFE UNIT
UNIVERSITY OF HELSINKI

DATE: December 7th, 2023

TO: Dr. Anne C. Hart
Academic Editor, PLOS Genetics
and
Dr. Gregory P. Copenhaver
Editor-in-Chief, PLOS Genetics

RE: Revisions on Verbeeren et al. PGENETICS-D-23-00671R1

We express our gratitude for the constructive review of our manuscript. We share the reviewers' assessment that this process has led to a much-improved paper. As per request by the Academic Editor, we have made the following adjustments to the paper:

- gene names are no longer capitalized in the manuscript
- we have now clarified that integrated strains were backcrossed at least five times with the wt strain
- integrated array nomenclature has been corrected
- we now have included all strains mentioned in the Materials and Methods in S1 appendix

In addition, we have included the access to the GEO data associated with the manuscript. The GEO reference series number is GSE249410 and includes accession numbers GSE249406, GSE249407, and GSE249408. To review GSE249410: go to https://www.ncbi.nlm.nih.gov/geo/query/acc.cgi?acc=GSE249410 and enter the token **azobyykqhtatnmn** into the box.

Kindly note that, upon request of reviewer 1, we have changed the title of our manuscript, which is now "The Muscleblind-like protein MBL-1 regulates microRNA expression in *Caenorhabditis elegans* through an evolutionarily conserved autoregulatory mechanism", with the accompanying short title "Autoregulation of *mbl-1* controls microRNA expression". In addition, we have reviewed our reference list and included one relevant reference [51] to the manuscript.

Responses to the reviewer's comments are listed below.

Reviewer 1: 1. "I am glad to see the new data on evolutionary conservation of the alternative exon. I'd still personally prefer the use of "ancient" to be changed to something more objective such as "evolutionarily conserved"."

We have accordingly avoided the use of "ancient" and replaced it with "evolutionarily conserved" in both the title and throughout the main text of the manuscript.

2. "The authors mention in the response to reviewers that it remains unknown which isoform of mbl-1 is predominant in touch neurons, but this is not mentioned in the text, as

far as I can tell. It would be useful to add to the text in order to appropriately caveat the interpretation of the neurite outgrowth assays."

Upon the reviewer's request, we have now included in the discussion section that we were unable to identify MBL-1 isoform preference in the TRNs and connect isoform expression with the TRN receptive field mutant results. We agree that it is important to clarify this point and thus it is an important addition to the manuscript.

3. "The text cites the literature that "…MBL-1 severely reduces lifespan," but the cited references [37, 46] demonstrate mild lifespan reduction (OP50) or more severe reduction (HT115). As such a modification of the text to "…MBL-1 severely reduces lifespan when grown on HT115 bacteria" or simply "…MBL-1 reduces lifespan" seem more appropriate."

The reviewer makes a fair point, and we have modified the text to simply state that "MBL-1 reduces lifespan".

We also want to reiterate that we now provide access to the GEO data associated with the manuscript. To review: please go to https://www.ncbi.nlm.nih.gov/geo/query/acc.cgi?acc=GSE249410 **and enter token azobyykqhtatnmn into the box.**

Reviewer 2: "I think the authors did a great job addressing the different comments and recommendations I made and have no further comments."

We thank the reviewer for the valuable contribution made to the manuscript.

Reviewer 3: "In this latest version of this manuscript, the authors have made an (honestly) impressive effort to address the reviewers' comments. This included a plethora or changes including to the introduction, methods, results, and discussion. Basically, every aspect of this manuscript has improved. While not every criticism resulted in a change (most did), all were addressed and the remaining criticisms are considered (by this reviewer) as a difference of opinion that do not preclude accepting the manuscript. I think, and hope the authors agree, this is a vastly improved effort."

We express our gratitude to the reviewer and agree that the comments have strengthened the overall quality of the paper.

Best regards.
Yours sincerely,

Susana Maria D.A. Garcia

Susana Garcia, Ph.D.

Institute of Biotechnolog
University of Helsinki
Biocenter 2, room 4023
Viikinkaari 5D,
00790 Helsinki, Finland

Phone: +358 050 4489545
Email: susanamaria.garcia@helsinki.fi
