# Supplementary material for: The Muscleblind-like protein MBL-1 regulates microRNA expression in Caenorhabditis elegans through an evolutionarily conserved autoregulatory mechanism
Source: PLoS Genet. 2023 Dec 22;19(12):e1011109. doi: 10.1371/journal.pgen.1011109 (PMC10773944; doi:10.1371/journal.pgen.1011109)
Supplement: Attachment — Submitted filename: PLoS_Genetics_02_Review.docx [file pgen.1011109.s026.docx]

This manuscript presents some very interesting observations on the autoregulation and function of the RNA binding protein MBL-1 in *C. elegans*. I deem it to be impactful and worthy of publication, provided that a few important but straightforward additions can be made:

1. Page 6: I feel that the point about the ancient evolutionary conservation is not made with appropriate depth, given that it is highlighted prominently in both the title and introduction. Some important analysis is included in the supplemental figures, but scarcely mentioned in the text (and not until the discussion), and thus should be amplified in the results section.
   a. Do the KR motifs show higher/lower/equal metazoan conservation when compared to critical conserved protein domains, *e.g.* MBL-1’s zinc finger domains, or other highly conserved motifs?
   b. Are the KR motifs subject to alternative splicing in similar ways throughout metazoa? If so, do the alternative exons tend to have the same boundaries, or does alternative exon size vary?
2. Line 199: does *mbl-1* mutation cause strong or weak defects in the *mbl-1* splicing reporter? Does it cause defects in one particular cell type? From what I can make of Figure S5 focusing on the excretory cell, it does not seem to any effect, as opposed to the strong effect observed via rt-pcr in Figure 1. Why would this be?
3. Line 238: which isoform of *mbl-1* is expressed in touch neurons? I can’t make them out in the micrographs. Does the isoform expression agree with the touch neuron receptive field mutant results?
4. Line 239: data is shown indicating that the two *mbl-1* isoform-specific mutants do not have defects in touch sensitivity. However, this is not highlighted in the text. It is important to disclose this in the text so as not to over-claim the findings about isoform-specific function.
5. Lines 303 &c: What is going on with all the genes dysregulated in the “short only” *mbl-1* mutants? According to the interpretation presented in the manuscript, one would expect this mutant to have roughly wild-type gene expression changes, but in fact there are roughly as many dysregulated genes as in the loss-of-function. This phenomenon warrants some initial characterization, and a mention in the text (*e.g.* are many of these transcripts directly bound by MBL-1? Possibly both direct and indirect targets?)
6. Line 443. I am curious about the varying lifespan results arrived at over the last few years for various *mbl-1* mutants*.* In this manuscript a large lifespan decrease is observed for a *tm1563* deletion or [*long only*] alleles. My lab previously observed a modest-to-no defect using deletion alleles *wy560* and *csb30* (PMID 28718764 and PMID 37214828) and the authors’ recent manuscript (PMID 34849877) found modest lifespan defects for *tm1563* with when grown on HT115 bacteria but no lifespan defect when grown on standard OP50. I hope the authors can expand on this, and for my own edification I’d be interested in any thoughts on the discrepancy, especially the authors’ own studies that both use the *tm1563* allele.

Minor point - Line 35: CRISP -> CRISPR

Other – I could not find links to the underlying sequencing data (*e.g.* GEO or SRA links)
