# Supplementary material for: The Muscleblind-like protein MBL-1 regulates microRNA expression in Caenorhabditis elegans through an evolutionarily conserved autoregulatory mechanism
Source: PLoS Genet. 2023 Dec 22;19(12):e1011109. doi: 10.1371/journal.pgen.1011109 (PMC10773944; doi:10.1371/journal.pgen.1011109)
Supplement: Attachment — Submitted filename: Response to reviewers.docx [file pgen.1011109.s027.docx]

PLoS Genetics PGENETICS-D-23-00671
Authors response to reviewers’ comments:

**Reviewer #1:**

1) **Although indicating that the manuscript presented “very interesting observations on the autoregulation and function of the RNA binding protein MBL-1 in *C. elegans*”, the reviewer also indicated that “the point about the ancient evolutionary conservation is not made with appropriate depth (…) and thus should be amplified in the results section.**”

As suggested by the reviewer, we have expanded our evolutionary analysis of the KR motifs (and the exons that encode these motifs), tandem Zinc finger motifs, and splice site signals/sequences of *mbl-1* isoforms (Figs 1B, S1, S2, S3, and S4). This analysis has been included in section 1 of the results (116 to 134).

**The reviewer further asked:**

a. **Do the KR motifs show higher/lower/equal metazoan conservation when compared to critical conserved protein domains, e.g. MBL-1’s zinc finger domains, or other highly conserved motifs?**

To address these questions, we performed an evolutionary comparison of the KR motifs vs the tandem zinc finger domains of MBL-1, and these results have been included in the manuscript (Fig 1B and Fig S4) and discussed in section 1 of the Results.

b. **Are the KR motifs subject to alternative splicing in similar ways throughout metazoa? If so, do the alternative exons tend to have the same boundaries, or does alternative exon size vary?**

As suggested by the reviewer, and to address these questions, we expanded the evolutionary analysis of the KR motifs in Bilateria and added the data to Figure 1B in the Results section. This figure features exon/KR motif alignments in representative Bilaterian species showing limited evolutionary conservation and varying exon sizes. S3 Fig further summarizes our findings regarding KR-motif associated alternative splicing in Metazoa.

2) **The reviewer asked “does mbl-1 mutation cause strong or weak defects in the mbl-1 splicing reporter? Does it cause defects in one particular cell type? From what I can make of Figure S5 focusing on the excretory cell, it does not seem to any effect, as opposed to the strong effect observed via rt-pcr in Figure 1. Why would this be?”**

The *mbl-1* mutation causes significant effects in the *mbl-1* splicing reporter, and these affect different tissues. An example is shown in Figs 4E and F where the presence of the *mbl-1* mutation causes a shift towards exon inclusion (increase in GFP signal) in the VNC, as would be expected due to *mbl-1* autoregulation. Similar effects are observed in the ganglia, in the head and the tail. In addition, we observed a developmental delay in neuronal cell bodies, whose effects we did not quantify. We further provide evidence that the absence of an effect on the excretory canal cell is due to FOX-1 compensating for the lack of MBL-1 in the mutant, and co-regulating AS of *mbl-1* (Figs 2F and G).

However, we understand the reviewer’s confusion with Figure S5 and the lack of effect in neuronal tissues. This lack of signal results from the insufficient efficiency of the RNAi. RNAi downregulation varies in a tissue-dependent manner, with neuronal tissues (CNS) known to be refractory to RNAi. This is the reason why the effect of the *mbl-1* mutation is more visible in Fig 4E, quantified in Fig 4F. Because it is redundant, and to avoid further confusion since this same issue has been raised by two reviewers, in the updated manuscript we have excluded the original S5 Fig.

3) **The reviewer asked “which isoform of mbl-1 is expressed in touch neurons?” and “Does the isoform expression agree with the touch neuron receptive field mutant results?”**

Unfortunately, it seems that *mbl-1* expression level in the touch neurons is below the detection level for our splicing reporters. This seemed to be independent of the promoter we used (S5 Fig). We therefore were unable to identify if there is a preference for isoform expression or connect isoform expression with the mutant results.

4) **The reviewer pointed out that “data is shown indicating that the two mbl-1 isoform-specific mutants do not have defects in touch sensitivity. However, this is not highlighted in the text. It is important to disclose this in the text so as not to over-claim the findings about isoform-specific function.”**

We have now added this clarification in the text, indicating that both isoform-specific mutants do not have defects in touch sensitivity (lines 270 and 271).

5) **Lines 303 &c: What is going on with all the genes dysregulated in the “short only” mbl-1 mutants? According to the interpretation presented in the manuscript, one would expect this mutant to have roughly wild-type gene expression changes, but in fact there are roughly as many dysregulated genes as in the loss-of-function. This phenomenon warrants some initial characterization, and a mention in the text (e.g. are many of these transcripts directly bound by MBL-1? Possibly both direct and indirect targets?)**

The reviewer raises a good point and one that we have tried to clarify in the text. Though both significance and fold change are lower, our analysis still indicate 860 dysregulated genes in the *short^(ex7-)^* *mbl-1* mutants. Interestingly, most of those genes show upregulation. Precocious or enhanced expression of the *short^(ex7-)^* *mbl-1* mutant therefore leads to the upregulation of these genes. It is not clear through which mechanism this occurs, whether the short^(ex7-)^ MBL-1 directly binds these targets and stabilizes the mRNA, or through an indirect mechanism. This is now included in the result section (lines 358 to 363) as well as in the discussion section (lines 533 to 541).

6) **The reviewer indicated that “I am curious about the varying lifespan results arrived at over the last few years for various mbl-1 mutants. In this manuscript a large lifespan decrease is observed for a tm1563 deletion or [long only] alleles. My lab previously observed a modest-to-no defect using deletion alleles wy560 and csb30 (PMID 28718764 and PMID 37214828) and the authors’ recent manuscript (PMID 34849877) found modest lifespan defects for tm1563 with when grown on HT115 bacteria but no lifespan defect when grown on standard OP50. I hope the authors can expand on this, and for my own edification I’d be interested in any thoughts on the discrepancy, especially the authors’ own studies that both use the tm1563 allele.”**

The reviewer raises and interesting point. Wang et al., 2008 (PMID: 18082131) found a significant decrease in lifespan with RNAi against *mbl-1* (HT115 bacteria). A similar observation was made in Sasagawa et al. (PMID: 19021294) with the *mbl-1(tm1563)* mutant on OP50 (p-value= 0.01). Our earlier work (PMID 34849877) with the *mbl-1(tm1563)* mutant confirmed the shorter lifespan both on OP50 and HT115 (p-value < 0.01; Supplementary Tables S1 and S2 in PMID 34849877), though the effect with HT115 is more drastic. Of note, our mutants were regularly outcrossed with the wild-type strain to prevent compensatory mutations affecting lifespan. The reviewer´s own work was done with deletion mutants grown on OP50 but did not find a significant decrease (PMID 28718764 and PMID 37214828). It seems that the discrepancy potentially results from the use of HT115 vs OP50 as bacterial food source. In their study of the impact of bacterial food sources on health span, Stuhr and Curran have shown that wild-type animals grown on HT115 live significantly longer than those grown on OP50 (PMID: 33159120). Moreover, the metabolic profiles of worms grown on OP50 and HT115 differ (PMID 28622510). It is possible that the microbiome modulates expression of MBL-1 or the MBL-1 regulatory network with an effect on lifespan, which could explain the more severe lifespan reduction with HT115. In the context of these differences, we found the work of Revtovich et al., 2019 (PMID: 30865620) relevant, calling for a consistent, defined diet to improve research comparisons.

**Minor points**

7) **Substitute the following: CRISP -> CRISPR**

This has been corrected in the text.

8) **Other – I could not find links to the underlying sequencing data (e.g. GEO or SRA links)**

We are currently in the process of submitting our data to the GEO repository.

**Reviewer #2:**

**1.- The reviewer asks to “clarify whether "replicated repeats" in different places in the manuscript refer to technical replicates (e.g., three RT-PCR from the same RNA extracts) or experimental replicates (RT-PCRs performed with RNA extracts from three independent biological materials).”**

As requested by the reviewer, we have clarified both in the figure legends and Materials and Methods that these “replicated repeats” correspond to biological repeats (from RNAs derived from independent samples). In addition, we further describe how three technical repeats were performed for each biological ‘repeat/sample’ used to generate qPCR data (Figs 1E, 5D, S10). Overall, throughout the manuscript, we now have included more detailed information on the sample size and statistical methods used for analyzing our data sets (Figs 2D, 2E, 2G, 3C, 3D, 3F, 4C and 4F).

**2.- Line 144 indicates "under both promoter 1 and 2", but it is unclear along the manuscript which of the two promoters is shown every time or whether both promoters are included in the same construct or other possibilities. Please clarify.**

We agree with the reviewer that the description of the usage of the promoters 1 and 2 was not clear. We have taken the following steps to clarify: we changed the text to make it clear that the isoforms are expressed under both promoter 1 and 2 *together* (line 154)*.* We have also modified Fig 1C to facilitate the reader´s interpretation. In summary, for our initial analysis to study the localization of the distinct MBL-1 isoforms, we used both promoters together to drive expression. We compared of tissue expression when using either promoter 1 or 2 (Fig S5). We decided to use promoter 1 only for the splicing analysis since expression showed a significant overlap between the two promoters but promoter 1 drives expression in a wider range of tissues (and for the sake of simplicity: to avoid co-expression of four constructs in the splicing reporter). We mentioned this section 2 of the results (lines 180 to 183). This promoter has also been used to express MBL-1 and rescue a synapse defect in PMID 22314215. We think that these modifications have improved interpretation of the results.

**3.- The reviewer stated that “I´d like the authors to reason why they conclude Mbnl1 short is "required", instead of "sufficient", for normal lifespan. Because in their manipulation they remove the Mbl1 long and maintain the short isoform, it is indeed difficult to classify whether this is a loss or gain of function evidence. However, considering "required/necessary" as an interpretation of experiments where you remove a causal element and "sufficient" when you put back in the system that element, I think it´d be more appropriate to conclude that Mbl1-short is sufficient for normal lifespan (and that Mbl-long is not required, if authors want).**

We agree with the reviewer that the isoform-specific deletion mutants constitute a special case that can be interpreted as both a loss or gain of function. For animals expressing the short^(ex7-)^ isoforms only, a gain of function interpretation indeed would identify these isoforms as “sufficient” for normal lifespan, whereas loss of function interpretation regarding the long^(ex7+)^ isoforms would identify these as “not required” for normal lifespan (as its absence has no effect on lifespan). For animals expressing the long^(ex7+)^ isoforms only, however, loss of function interpretation for the short^(ex7-)^ isoforms identifies these short^(ex7-)^ isoforms as “required” for normal lifespan (its absence reduces lifespan to that of the *mbl-1* mutant). Taken both mutants and the lifespan results into account, we argue that expression of the short^(ex7-)^ isoforms can be interpreted both to be sufficient and required for normal lifespan. Because of the apparent and potential misinterpretation of the sufficiency condition (PMID: [29757057](https://pubmed.ncbi.nlm.nih.gov/29757057)), we find the use of “required” more informative. However, should the reviewer deem our logic flawed, we are open to use “sufficient” instead.

**4.- The reviewer asks that “Upon CRISPR editing, authors must compare Mbl1 levels previous to the manipulation and after. This is to rule out potential overexpression effects associated to the manipulation and assign phenotypes exclusively to the loss of function of the missing isoform. This is, can the authors confirm no significant changes in protein expression after generating Mbl-1 short and long, and after other genetic manipulations that introduced reporters”?**

Following the reviewer’s point, we have now compared *mbl-1* mRNA transcript levels in all the different CRISPR-edited mutants with qPCR and found no significant differences (Fig S10).

**5.- The existence of two Muscleblind promoters was also previously reported for Drosophila and humans (e.g.** [**https://journals.plos.org/plosone/article?id=10.1371/journal.pone.0093125**](https://journals.plos.org/plosone/article?id=10.1371/journal.pone.0093125)**), perhaps worth mentioning in the context of phylogenetic conservation.**

We agree with the reviewer’s suggestion. Therefore, in the updated version of the manuscript we have expanded on the phylogenetic conservation of *mbl-1* by: analyzing the phylogenetic conservation of the alternative *mbl-1* promoters (Fig S1), including a reference to *Drosophila* and human promoters, and exploring the functional relevance of the use of the alternative promoters in the Discussion section (in addition to the potential significance of other MBL-1 isoforms) (lines 542 to 553).

**6.- It is unclear whether additional Mbl-1 isoforms exist in C. elegans or if these two are the only ones. Please clarify and discuss.**

Reviewer #3 also raised a similar point. To address this, we have greatly expanded our analysis to detect additional *mbl-1* mRNA isoforms. We performed long RT-PCR resulting in the detection of eight distinct isoforms, whose data have been included (Fig 1A). These data have led to a significant restructuring of the Results section 1, as well as the inclusion of the phylogenetic analysis of these isoforms. This analysis led to the conclusion that our previous use of simply “short” and “long” *mbl-1* isoforms was an inappropriate oversimplification. Therefore, we have modified the text and termed the isoforms that include exon 7 as long^(ex7+)^, while the isoforms that skip exon 7 are now named short^(ex7-)^. In our opinion, this is a more accurate reflection of the fact that exon 7 inclusion/exclusion, as well as the effect it has on protein localization/functionality, is the main determinant of the phenotypes we describe in the paper. As mentioned above, the potential significance of the MBL-1 isoforms is considered in the Discussion section (lines 542 to 553).

**Minor issues
1.- note the current name for MBNL:** [**https://www.ncbi.nlm.nih.gov/gene/4154**](https://www.ncbi.nlm.nih.gov/gene/4154)

As suggested by the reviewer, we have included the current name for MBNL. However, we would like to mention that we feel that the term “splicing regulator” is too narrow, and downplays MBNL’s wide range of functionalities not related to splicing. However, we have used the term “splicing regulator” when we introduce the human MBNL1 gene, since it is its official name. In other contexts, we use “muscleblind-like protein” similarly as in: <https://www.ncbi.nlm.nih.gov/gene/4154> .

**2.- Myotonic dystrophy type 1 and 2 is far more common than with Roman numerals I and II**

As suggested, this has been changed.

**3.- Use "Student´s"**

We have corrected where necessary.

**Reviewer #3:**

1) **The reviewer “struggled to understand some of the study design. Some of the molecular manipulations were unclear to me. For example, the study assumes the existence of only two mbl-1 isoforms despite the literature evidence to the contrary. In figure 1 two promoters are shown for a long and a short isoform of mbl-1. It was not immediately obvious if the use of Promoter 2 always resulted in a long isoform including exon 7, and the use of Promoter 1 always resulted in a shorter isoform lacking it. The diagrams in the figures did not really help me understand this. Particularly in figure 1 where in B two promoters (1 and 2) are shown driving expression of the mCherry reporter.”**

A similar comment was made by Reviewer #2 and thus we understand the Reviewer’s frustration and agree that the study design was not described clearly. We have updated the text and changed the diagram to clarify the point that expression of each isoform was driven by promoter 1 and 2 *together*, through simultaneous co-injection of two constructs (Fig 1C). Also, the reviewer is correct in that more than two *mbl-1* isoforms are expressed, and we performed long RT-PCR resulting in the detection of eight distinct isoforms (Fig 1A). We included a schematic overview of the detected *mbl-1* isoforms. We are confident that these changes will improve the reader’s interpretation of the experiments performed. In addition, we compared *mbl-1* expression patterns under each of the two promoters (Fig S5).

**The reviewer adds that “a much cleaner experiment would have been to use a null mbl-1 mutant that removes all known mbl-1 isoforms (such as mbl-1(wy560) reported by Spilker et al.), and then introduce the mCherry tagged rescue constructs into that strain. This would have allowed the authors to make phenotypic assignments without worrying about alternative isoform expression.”**

We understand the reviewer’s rationale; however, our preference for the use of *mbl-1(tm1563)* results from the fact that *tm1563* is effectively a loss-of-function mutant. Also, the genetic mutation wy560, in addition to removing all *mbl-1* isoforms, also deletes an additional 7 genes (PMID:22314215), raising the issue of possible *non-specific* phenotypes. Finally, to facility the interpretation of the experimental setup and the results, we have made changes to the diagram and in text: our goal with the mCherry constructs was to evaluate the localization of MBL-1 isoforms that either include or lack exon 7.

**The reviewer further adds that “because the coding sequence for the longest mbl-1 isoform is still relatively short (<3kb), it would have been possible for the authors to investigate the features of the KB region in exon 7 responsible for nuclear localization. This could be done relatively easily using even an extrachromosomal array to drive the expression of a GFP-tagged MBL-1 isoform where site-directed mutagenesis was used to mutate the KR motif or other (control) exonic regions. That would have significantly improved the resolution power of the study.”**

We concede that the features of the KR motif (and that of other regions) could be evaluated through targeted site-directed mutagenesis with such construct. The reviewer suggests that such an experiment would enable us to study the requirement of the KR motif (or other sequences on exon 7) for nuclear localization and/or functional assignment/phenotype rescue studies.

We would like to note that evolutionary conservation of KR function in MBNL nuclear localization has been previously demonstrated: site-directed mutagenesis of the KR motif in *Drosophila* Muscleblind protein results in reduction of nuclear localization in mammalian (PMID: 18286170) and *Drosophila* cell lines (PMID: 20652497). Similarly, KR motif mutagenesis in human MBNL disrupts nuclear localization (PMID: 25274774). We therefore deem it reasonable to assume that the presence of the double KR motif serves a similar role in *C. elegans* MBL-1, though we have been careful to avoid stating this as such, and rather use wording such as “inclusion of exon 7 leads to nuclear localization”.

**The reviewer also asks “the CRISPR/Cas9 strains that are reported to exclusively express either long or short isoforms are reported in the methods to have been outcrossed at least 5 times with N2 animals. Was the genotype of the resulting crosses confirmed through sequencing.”**

After initial genotype conformation of the CRISPR/Cas9 strains through sequencing, genotypes resulting from crosses were confirmed through a PCR strategy enabling the unique identification of the mutant (distinguishing between wt, heterozygote, and mutant). PCR primers used for genotyping have been added to the primer (S4 appendix).

**2) Context of the study: There is some discrepancy on the literature with regards to the actual number of mbl-1 isoforms. While the authors cite these studies, they make no mention of this discrepancy nor assess the impact on their results. For example, Wang et al (2007) reported two mbl-1 isoforms, but later work by others (i.e. Segawa et al., 2009, Spilker et al., 2012) reported six isoforms of mbl-1. This is further (and more recently) supported by the CenGen isoform analysis (with CENGEN actually providing evidence for eight isoforms,** [**http://splicing.cengen.org/gene/WBGene00019347/**](http://splicing.cengen.org/gene/WBGene00019347/)**). This is a rather important detail to leave out because there is no telling how these additional isoforms might impact the experiments performed or the results observed.**

The reviewer highlights a valid point with the prevalence of some ‘confusion’ in the field relative to the number of the *mbl-1* isoforms. We have updated our results section to mention the six alternative transcripts reported by Sasagawa et al., 2009, which are the same as in Spilker et al. 2012. It is important to note that these alternative transcripts differ only through their use of an alternative promoter site, and in the inclusion/exclusion of exon 7, both under investigation in our paper. One of the isoforms described in these papers skips the whole of exons 4, 5, 6, and 7, and is most likely the result of splicing noise since this splicing event is not categorized in CENGEN, we could not detect it in our analyses, and only a few reads were found from a quantitative RNA-seq meta-analysis using more than 1600 RNA-seq data sets (PMID: 29089372).

We carefully considered the point made by the reviewer, and significantly restructured the result’s section 1 to reflect the existence of additional isoforms, and to include a more thorough phylogenetic analysis of *mbl-1* (as requested by Reviewer #1). As mentioned before, our long RT-PCR analysis identified a total of eight isoforms (Fig 1A), and we included the discussion of this analysis in the text. We believe that our previous assessment of *mbl-1* isoforms as simply “short” and “long” was an inappropriate oversimplification, and we have changed those terms in the text so that isoforms that include exon 7 are referred as long^(ex7+)^, while isoforms that skip exon 7 are now named short^(ex7-)^. In our opinion, this is a more accurate reflection of the fact that exon 7 inclusion/exclusion is the main determinant of the phenotypes we describe in the paper. It also reflects the effect inclusion/exclusion has on protein localization/functionality (as stated in our reply to Reviewer #2). Considering the presence of these additional isoforms, we have changed the discussion section to provide alternative/additional interpretations of our experimental results.

We thank the reviewer for bringing the CENGEN database to our attention. The reviewer mentioned eight isoforms, yet further inspection of the data revealed instead evidence for eight alternative “splicing events”, identified through RNA-seq splice junction reads. Here, I will describe and provide a putative assessment of these splicing events:

- AS event 1 provides evidence of yet another upstream located promoter, albeit used very rarely (<< 1%).
- AS events 2, 3, and 4 relate to intron retention events that are very rare (<< 1%), and almost certainly the result of splicing noise since inclusion would lead to transcripts that are NMD targets.
- AS events 5, 6 and 7 relate to the inclusion/exclusion of “exon 8” and “exon 9” (“exon 7” in our paper). AS of “exon 8” is very rare, according to both the CENGEN project and PMID: 29089372 (we could not find any evidence for it from our routine PCR). Furthermore, both its splice site signals and encoded AA sequence are not conserved in nematode species (see also Fig S8), further calling its functional relevance into question. AS of exon 9 (our exon 7) is functionally conserved and is the key splicing event discussed in our paper.
- Finally, AS event 8 relates to a regulated intron retention event. This results in the formation of two isoforms for which the last nine amino acids differ, as well as the length and composition of the 3’UTR. This AS event occurs frequently (CENGEN and PMID: 29089372) and is conserved in nematodes (S2 Fig). Through PCR, we have identified this splicing event, evaluated its evolutionary conservation, and we discuss in the manuscript its potential functional relevance.

Thus, in our view, based on evolutionary conservation and frequency of occurrence, the following alternative mRNA processing mechanisms are of essence in the *mbl-1* gene:

- alternative promoter usage (promoter 1 vs promoter 2) with addition of extra AA residues at the N-terminus when promoter 2 is used.
- autoregulatory splicing of exon 7 with implications on protein localization and functionality.
- autoregulatory splicing leading to alternative polyadenylation. Transcripts have different 3’UTRs and proteins differ in the nine C-terminal AAs.

However, we cannot exclude that a rare isoform expressed under certain conditions or during a defined developmental stage exerts a crucial role, even in the absence of evolutionary conservation (a species-specific isoform). However, since splicing is an inherently noisy process and evolutionary conservation is the strongest indicator of function, we believe that the null hypothesis should be that these rare isoforms are non-functional unless proven otherwise.

**The reviewer further added “One example that comes to mind involves the mutants used. While the mutation in tm1563 results in an in-frame deletion for longer isoforms, for the three shorter isoforms that start in exon-3 the mutation results in a frame-shift. This means that this mutation does not affect all available isoforms the same way.”**

Sasagawa et al. reports a deletion of 512 bp for mutant strain *tm1563*, which eliminates exon 3 and the transcription start site used by promoter 1. Exon 3 has a length of 121 bp and its exclusion leads to a frame shift. This causes an out-of-frame deletion rather than in-frame, resulting in a premature stop codon for all isoforms, regardless of which start codon AUG is used. Therefore, all generated isoforms are out of frame, rendering the *mbl-1(tm1563)* strain effectively a loss-of-function mutant. To avoid any confusion about this mutation and the reason why we chose to use it, we have clarified this issue in results section 1 of the manuscript (lines 165-166).

**3) The reviewer pointed out “At times the authors test and compare L3 larva, other times L4, yet other times Day-1 adults (e.g. behavioral assays). I think that the study could be improved by selecting and testing a single stage, or alternatively reporting their findings for all stages. If fluorescence microscopy limits comparison to L3 animals, then this stage should be compared throughout (e.g. behavioral tests, qPCR, etc). The present approach clouds interpretation of the results (at least those involving comparisons across life stages).”**

We agree with the reviewer that our studies would be improved by the ability to compare the different assays at one particular stage, or alternatively all stages. To address that we have now repeated, or performed additional analysis, for all behavioral assays (Figs 2C, 2D, 2E and 2F: thrashing assay, soft touch stimulation assay) and fluorescence microscopy (Figs 2F, 2G, 4E and 4F) with L4 animals. Thus, all experiments have now been conducted testing a single stage (qPCR, RNA-seq, RIP-seq, miRNA-seq, behavioral assay, splicing reporter microscopy), facilitating comparison and interpretation.

**The reviewer further points out that “In figure 1 we see mbl-1 expression in head and tail ganglia, ventral nerve chord, and spermatheca, but in the alternative splicing reporters is seen in ventral nerve chord but not in the head or tail ganglia. How do the authors account for this differential expression?”**

We agree with reviewer that, in the image shown, *mbl-1*’s expression in the head and tail glia was not discernable when using the alternative splicing reporter. To make *mbl-1*’s expression pattern clearer, we have provided additional and clear images of the alternative splicing reporter during development from the L1 to the L4 stage (Fig 2B). Expression can now be discerned in the head or tail ganglia. We also refer to S10 Fig for expression of the alternative splicing reporter at the L4 stage under promotor 1 and 2, respectively.

**4) RNAi experiments: With regards to the inactivation of fox-1 through RNAi: was this done in a neuronally sensitized strain? It is known that many tissues in C. elegans are refractory to RNA interference and require the ectopic expression of a double stranded RNA channel to be successfully silenced. Because of this, silencing of fox-1 or any other gene in the absence of a sensitized background could only be expected to alter expression in tissues that are non-refractory to RNAi (e.g. glia and muscles) but not in neural tissues.**

The RNAi experiment was not performed in a neuronally sensitized strain. The reviewer raises a fair point about certain tissues being refractory to RNAi, most notably neural tissues. Since our primary focus with this experiment was the excretory canal cell and the role of FOX-1 on AS of *mbl-1* in this tissue, we did not consider other tissues. Note that in the updated manuscript we have excluded the original S5 fig from the paper since it was redundant with regards to the role of FOX-1 on *mbl-1* AS in the excretory canal cell, and for the reasons properly pointed out by the reviewer.

**5) The reviewer asks: for the “Behavioral assays: what is the estimated ISI for the repeated touch stimulation and how this compares to the results in the literature? Was ISI kept to 30 sec, 60 sec, etc.?”**

Our touch stimulation assay was done as originally described in Chalfie and Sulston, 1981 (PMID: 7227647), and more specifically Hobert al., 1999 (PMID: 9885243). This method does not stipulate an ISI, but rather is based on the rapid assessment of touch responsiveness by 10 alternating strokes across the anterior and posterior half of the nematode. We have now included these references in the Materials and Methods section. Experiments from Hobert et al., 1999 (adult animals) and Sarasija et al., 2018 (PMID: 29989545) (L4 stage and adult animals) show ca. 90% (adult), 87% (L4) and 82% (day 1 adults) touch responsiveness. In our hands, wild-type animals showed an average touch responsiveness of ca. 78% (with high reproducibility from three independent repeats: Fig. S5 Appendix). Note that such experiments often include variability in forces due to differences in mechanical properties of hair used or the performance of the experimenter as studied in Nekimken et al., 2017 (PMID: 28542494).

**The reviewer also asked “What frame rate and magnification was used for the swimming movies.”**

In our swimming movies, we used 10x magnification and 80 frames/s frame rate. We have updated the manuscript and included this information in the Materials and Methods section.

**The phenotypes investigated were also vague or generic and do not provide opportunities to generate mechanistic insights with regards to the isoforms function. For example, just about any deleterious mutation or manipulation could be predicted to affect an animal’s lifespan or motility. Going after more specific phenotypes would have been more informative in this part of the study.**

We understand the reviewer’s comments on the generality of the genotypes under investigation. Our aim was to uncover cell-type- or tissue-specific differences in MBL-1 isoform regulation and investigate these tissues with relevant assays. This proved to be non-trivial. Interestingly, the reviewer has brought the CENGEN database to our attention, which has *mbl-1* AS information on the single-neural cell level. This information can be used in future studies together with our splicing reporter to discover co-regulators, as well as to assess isoform function at single cell level.

The reviewer also mentioned the fact that deleterious mutations, or manipulations can affect lifespan or motility. We agree with the reviewer that that does not mean that that gene regulates (i.e. is instructive for) the phenotype in question. The distinguishing feature of MBL-1 is that the effect on phenotype depends on the isoform in question. For all the phenotypes under investigation, the long MBL-1^(ex7+)^ isoforms seem to be dispensable (i.e. not permissive). This puts in focus the short^(ex7-)^ isoforms and its requirement for normal lifespan (possibly through the ability to regulate miRNAs), neuromuscular functioning and neurite outgrowth processes (possibly through regulation of transcripts coding for cytoskeletal components).

**6) Longevity study: With regards to the study of longevity in C. elegans using FUDR, its use has recently been challenged by the finding that 5-fluorouracil itself has complex effects on lifespan which can cloud the interpretation of results (DOI: 10.1266/ggs.15-00064). It would therefore be recommended to validate the main results without using FUDR to control for any potential artifacts.**

We understand the reviewer’s concern about the potential effect of 5-fluorouracil (5-FU) on lifespan.  An effect of 5-FU has been shown for mutants that interfere with DNA damage repair and fertility pathways. However, the study referenced by the reviewer (PMID: 27582048), in which lifespan was affected by an endonuclease mutant, states that: “FUdR is very useful when measuring worm lifespan. However, it may not be suitable for measuring the lifespan of mutants that show abnormal reproduction”. Our mutants do not show abnormal reproduction, nor could we infer from our RNA-seq dysregulation of DNA repair pathways. In addition, the concentration of 5-FU used in our study was 5-fold lower than the concentrations used in the assays described in PMID:27582048, further diluting any potential effect.

**Furthermore, was mRNA levels for the two isoforms quantified to control for dosage differences potentially driving longevity differences observed between the treatments?**

The mRNA transcript levels were compared in all the different CRISPR mutants with qPCR and we found no significant differences (S10 Fig).

**7) The reviewer indicates that “The authors used t-tests almost universally to compare all their data (except for the survival curves). However, t-tests require a very specific set of circumstances to be validly performed.” And expresses concern that “Throughout the study, it is clear that the authors used t-tests when they should have used ANOVAs, and there is to mention to performing tests of normality or equal variance.” In addition, the reviewer points out that “Sample sizes are not usually provided although, I eventually figured (from the appendix Table S6) that most comparisons involved samples of just ten animals (Figure 1D is an exception and no raw data or N is provided for these data).” The reviewer also asks whether “ all were measured during a single assay? (…) I would therefore recommend that the authors justify the sample size they used in the context of power statistics (the beta value for their comparisons) or in the context of similar studies in the field.” Finally the reviewer recommends that “If there are two or more manipulations (and there is one control group) the appropriate approach is to perform an ANOVA (if all groups are normally distributed and pass the equal variance test, or an ANOVA of ranks otherwise). Following the ANOVA, a Dunnett's test may be used for relevant treatments to be compared against a control group (N2s in this case).”**

We thank the reviewer for the thorough and careful critique of our experimental analysis. We have addressed these issues and significantly improved our statistical analysis. We have performed D’Agostino-Pearson test for normality and Bartlett’s test for equal variance prior to applying the appropriate test (ANOVAs and post-hoc tests: Tukey and Dunnett’s, Kruskal-Willis for nonparametric data and Dunn’s post-hoc test). We have also increased the number of animals in our assays by a substantial margin (both in behavioral assays as for fluorescence microscopy experiments) and performed assays with animals grown on independent plates to eliminate plate-specific variations. We included this information in both the figure legends and the Materials and Methods section of our manuscript. In addition, we added numerical values and information on independent, biological repeats in S5 Appendix.

**8) Figures: The figures of the study were not up to publication standard. For example, there is not a single scale bar in any of the confocal images shown. There is also no consistency in orientation or description of orientation. Animals are presented head to the left or right in the same figure and often ventral and dorsal directions are left unresolved. Many composite plates do not have sub-indexes (eg. i, ii, iii). However sometimes the authors use non-standard quotations (‘, ‘’, ‘’’).**

We have improved the quality and interpretability of the figures and the corresponding images. All of our images now feature the animals in comparable orientation along the anterior-posterior axis with indication of head and tail region. Dorsal and ventral sides are also indicated, and scale bars are provided. Multi-panel images are now properly numbered using a standard sub-indexes (I, II, III).

**The reviewer points out that “It is unclear what the justification for the variability in choice of error measurement reported throughout the study is. For example, in Figure 3C error bars represent 1.5 IQR but in Figure 3D and F error bars represent SEMs. In some figures data is presented as vertical bars with mean and SEM errors (Figure 1D), in other figures the authors use point plots and mean bars (Figure 2D E and G), other times they use box plots with IQR (Figure 3C), and yet other times they use violin plots (Figure 4C). The authors should stick to a consistent representation of similar data to avoid confusing the reader.”**

We have followed the reviewer’s recommendation, have standardized the choice of plots used and employed consistent representation of error bars (Figs 1E, 2D, 2E, 2G, 3C, 3D, 3F, 4C, 4F, and 5D).

**9) Discussion: In the context of DNA transcription and gene splicing I would not go as far as the authors to claim that the mechanism investigated displays “extreme” conservation. It might be conserved, but not any more conserved than other mechanisms associated with gene transcription and regulation. Much of the discussion was highly speculative and not directly supported by the data presented. Some of the interpretation was only supported in a tenuous manner. For example, the phenotypes investigated (e.g. lifespan and locomotion) are so broad that literally any insult to the organism would be predicted to affect them in these metrics. That does not mean that the insult is directly related or responsible for the phenotype. For example, one can predict that any illness might affect a person’s top swimming speed, be it a cold, Parkinson’s disease, arthritis, or a muscular sprain. This does not mean that a gene related to these diseases are also responsible for swimming in humans. Similarly, showing that sick worms swim slower or live less than healthy ones does not mean a gene is associated with longevity or the production of locomotion. That type of phenotypic assignment would require more narrowly defined parameters, and additional experimental manipulations.**

We edited the manuscript to address the reviewer’s concerns. We have avoided the use of words as “extreme”, though note that, unlike with gene regulation, conservation of functional alternative splicing that predate the split between vertebrates and invertebrates (670 million years ago) is indeed very rare with only 21 known cases (Gomez et al., 2021; PMID: 34302486)). We further carefully adjusted wording in our discussion to limit speculation, and separate speculation from clear statements, and attempted to avoid overinterpretation of our data. We re-iterate (and want to emphasize) the point about MBL-1 in that “the distinguishing feature with MBL-1, however, is that the effect on phenotype depends on the isoform in question”. For all the phenotypes under investigation, the long MBL-1^(ex7+)^ isoforms seem to be dispensable (i.e. not permissive), whereas short^(ex7-)^ isoform expression is required. By no means we would want to suggest that the short^(ex7-)^ MBL-1 isoforms directly regulate lifespan (or locomotion) as we believe that such a claim would have to be followed up by additional experiments (e.g. correlate dosage with phenotypic improvement). We therefore attempted to avoid any claims that could be interpreted as such in the manuscript.
